# Tau and tauopathies across primate species: implications for modeling neurodegenerative disorders

**DOI:** 10.3389/fnagi.2025.1598245

**Published:** 2025-07-23

**Authors:** Julia C. Colwell, Marina E. Emborg

**Affiliations:** ^1^Preclinical Parkinson’s Research Program, Wisconsin National Primate Research Center, University of Wisconsin, Madison, WI, United States; ^2^Cellular and Molecular Pathology Graduate Program, University of Wisconsin, Madison, WI, United States; ^3^Department of Medical Physics, University of Wisconsin, Madison, WI, United States

**Keywords:** tau, tauopathies, nonhuman primates, Alzheimer’s disease, frontemporal dementia, neurofibrillary tangles, aging, AT8

## Abstract

Tauopathies are neurodegenerative disorders characterized by the abnormal accumulation and aggregation of hyperphosphorylated tau protein. They can be primary or secondary depending on whether tau inclusions are the predominant pathology (e.g.: frontotemporal dementia related to tau) or are found with other proteinopathies (e.g.: Alzheimer’s disease), respectively. Currently, there are no effective treatments to prevent or slow down progressive tau accumulation. Animal models play a critical role in the efforts to unravel the mechanisms leading to tauopathies and identifying therapeutic targets. Nonhuman primates (NHPs) present several advantages for the study of tauopathies, as they have complex neuroanatomy and behavior that resembles human traits, and their tau gene and protein are highly conserved. Moreover, aged NHPs, like humans, can present various tau inclusions in their brains, although whether NHPs can develop human-like tau-related neurodegenerative disorders is currently debated. The main goal of this review is to analyze available reports on tau pathologies and models of tauopathies in NHPs considering the complexity of the tau protein and associated tau pathologies. Here, we first summarize current available information on human and NHP tau under physiological conditions in order to highlight species differences and gaps in knowledge. We then analyze reports on tau pathologies in aged NHPs compared to human aging and tauopathy, followed by an evaluation of current and emerging NHP models of tauopathy. Lastly, we discuss the practical and ethical challenges of doing tauopathy research in NHPs, and how to best leverage it to ultimately find solutions for patients with these disorders.

## 1 Introduction

Tau was first described in 1975 as a protein factor essential for self-assembly of tubulin dimers into microtubules (Weingarten et al., 1975). Today, tau is a notable member of the microtubule-associated protein (MAP) family (Dehmelt and Halpain, 2005). In the healthy adult central nervous system (CNS), tau is primarily associated to neuronal axons where it provides microtubule stability and regulates motor and vesicle transport (Lee and Leugers, 2012). Tau is mainly recognized for its role in neurodegenerative disorders termed “tauopathies,” in which hyperphosphorylated tau forms intracellular inclusions. While all tauopathies are defined by abnormal accumulation of phosphorylated tau (p-tau) and progressive neurological dysfunction, each disorder displays variations in clinical presentation, pathological tau conformation, and spatial–temporal tau accumulation. Tauopathies are classified as primary or secondary, depending on whether tau pathologies are the predominant features or they are present with other pathologies. Examples of primary tauopathies include sporadic and genetic forms of frontotemporal dementia (FTD) related to tau. Alzheimer’s disease (AD) is considered a secondary tauopathy, as beta amyloid (Aβ) plaque deposition precedes tau accumulation (Cody et al., 2024). Today, there are no effective treatments to prevent or slow down progressive p-tau accumulation.

Different animal models of tauopathies have been developed, including genetically modified rodents, fruit flies, worms, and zebrafish expressing gene mutations associated to tau pathology in humans (Dujardin et al., 2015). These models help to unravel tau-related mechanisms of disease, identify therapeutic targets, and evaluate novel therapies. Nonhuman primates (NHPs) have several species-specific advantages for the study of tauopathies. NHPs have complex neuroanatomy, including a cingulate gyrus with greater size caudally than rostrally and a reduced olfactory lobe and bulb (Gonzales et al., 2015). An enlarged frontal lobe allows NHPs to perform higher order executive functioning, which facilitates assessment of attention, learning, and memory (Phillips et al., 2014). Crucially, the *microtubule-associated protein tau (MAPT)* gene encoding for tau and the tau protein sequence are highly conserved between humans and NHPs, with increasing differences across the phylogenetic tree. Moreover, various tau inclusions can be present in the brain of aged NHPs, similar to aged humans (Walker and Jucker, 2017), although whether NHPs can develop human-like tau-related neurodegenerative disorders is currently debated.

The main goal of this review is to analyze available reports on tau pathologies and models of tauopathies in NHPs considering the complexity of the tau protein and associated tau pathologies. Here, we first summarize current available information on human and NHP tau under physiological conditions in order to highlight species differences and gaps in knowledge. We then analyze reports on tau pathologies in aged NHPs compared to human aging and tauopathy, followed by an evaluation of current and emerging NHP models of tauopathy. Lastly, we discuss the practical and ethical challenges of doing tauopathy research in NHPs, and how to best leverage it to ultimately find solutions for patients with these disorders.

## 2 *MAPT* gene and tau protein structure and function

In humans the *MAPT* gene is located on chromosome 17q21.1 and has two haplotypes, H1 and H2, which represent a ~1 MB inversion of one another (Zody et al., 2008). H1 homozygosity is more common in the human population and is associated with an increased risk for tauopathies, whereas H2 homozygosity seems to be neuroprotective (Wade-Martins, 2012).

**Figure 1:**
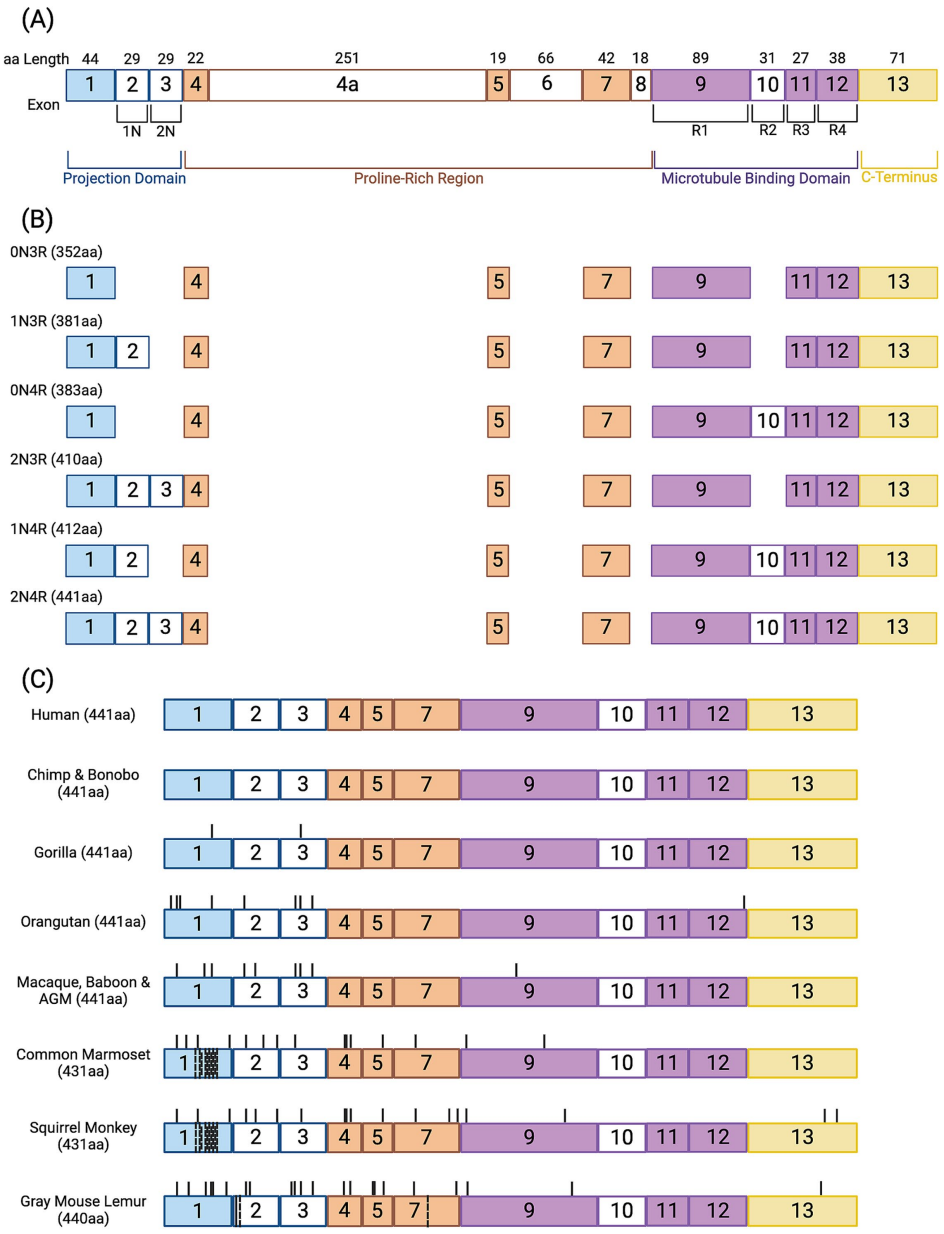
Tau protein in human and nonhuman primate species. **(A)** Tau has four distinct regions: (1) the N-terminal projection domain (blue outline), (2) the proline-rich region (orange outline), (3) the microtubule binding domain (purple outline), and (4) the C-terminus (yellow outline). Colored boxes represent constitutively expressed exons; empty boxes are exons subject to alternative splicing that translate into **(B)** the six CNS isoforms of the tau protein. Expression of exons 4a, 6, and 8 is restricted to the human PNS and retina. **(C)** The longest CNS tau isoform in primates is the 2N4R tau. Black ticks in the protein region identifies amino acid (aa) variations in 2N4R tau in each nonhuman primate species compared to the human sequence. Common marmosets, squirrel monkeys, and gray mouse lemur tau has aa deletions, indicated by dashed black lines through the exons in which they occur. Gray mouse lemurs have an aa addition in exon 2, denoted by a solid black line through the exon. Created in https://BioRender.com.

*MAPT* contains 16 exons, of which 8 are constitutively expressed (exons 1, 4, 5, 7, 9, 11, 12, and 13) (Figure 1A). Exons 2, 3, 4a, 6, 8, and 10 are subject to alternative splicing, and exons 0 and 14 are transcribed but not translated (Andreadis, 2005). The tau protein has four distinct regions: (1) the N-terminal projection domain, encoded by exons 1–3; (2) the proline rich region (PRR), encoded by exons 4–8; (3) the microtubule binding repeat (MTBR) region, encoded by exons 9–12; and (4) the C-terminal domain, encoded by exon 13. The human CNS expresses six distinct tau isoforms, depending on the combination of exons transcribed (Figure 1B). The isoforms differ by the number of N-terminal inserts and microtubule binding repeats (MTBR). Isoforms may contain zero (0 N), one (1 N), or two (2 N) N-terminal inserts; exons 2 and 3 encode 1 N and 2 N isoforms, respectively. Tau may contain either three (3R) or four (4R) MTBRs, differentiated by the inclusion of exon 10 (Andreadis, 2005; Andreadis, 2013).

The four regions of the tau protein direct its activity. N-terminal projection carries a negative charge which is repelled by the negatively charged tubulin surface. It contributes to the spatial organization of tau by extending out from the microtubule surface and interacting with the axonal plasma membrane (Amos, 2004; McKibben and Rhoades, 2019). Expression of exons 2 and 3 can negatively regulate microtubule binding through interactions with the PRR flanking the N-terminus immediately downstream. The PRR carries a net positive charge and can independently promote tubulin polymerization and enhance the microtubule binding ability of the MTBR (McKibben and Rhoades, 2019). The MTBR can have up to four 31–32 amino acid-long repeats, denoted as R1, R2, R3, and R4, respectively (Mandelkow et al., 2007). The MTBRs interact with the microtubule surface through weak van der Waals interactions and highly shielded ionic forces, and the inclusion of exon 10 (R2) strengthens this interaction (Goode and Feinstein, 1994). The C-terminus helps stabilize tau and prevent aggregation (Geng et al., 2015).

Tau protein expression changes across the human lifespan. In the fetal human CNS, all tau isoforms are 0N3R (Andreadis, 2005). Exon 10 inclusion increases dramatically during the perinatal period and persists throughout childhood and adult life. Exon 2 inclusion stably increases from the perinatal period until approximately 10 years of age, at which its expression plateaus. Exon 3 inclusion slightly increases from fetal to adult stages but remains at lower levels than exons 2 and 10 (Hefti et al., 2018; Takuma et al., 2003). Healthy adult human brains express 3R and 4R isoforms in an approximate 1:1 ratio. 0 N, 1 N, and 2 N isoforms comprise ~9%, ~37%, and ~54% of all adult CNS tau isoforms, respectively (Liu and Gong, 2008). Differences in *MAPT* splicing are minimal across neuroanatomical regions throughout the human lifespan (Hefti et al., 2018).

The few studies assessing expression of tau proteins across brain regions have focused on areas typically affected by AD. Total tau expression seems to be most abundant in the hippocampus and cortical gray matter (Paterno et al., 2022; Trojanowski et al., 1989). Tau truncated at the N- and C-terminus, which has been proposed to increase tau aggregation in AD (Gu et al., 2020), was found in the hippocampus, entorhinal, prefrontal, and motor cortices in adults ranging in age from 18–104 years, with more widespread C-terminal than N-terminal truncation. Notably, an age-dependent loss of full sequence tau associated to C-terminal truncation was observed in the entorhinal cortex, particularly in individuals aged >60 years (Friedrich et al., 2021).

Physiological tau is heat- and acid-stable, highly soluble, and lacks a higher order structure; thus, it is considered an intrinsically disordered protein (Mandelkow et al., 2007). Over half of the primary sequence is comprised of the amino acids Glycine (Gly), Lysine (Lys), Proline (Pro), Serine (Ser), and Threonine (Thr) residues, all of which except Thr are disorder-promoting (Mandelkow et al., 2007; Uversky, 2019). This primary structure elicits an extended, “random coil” secondary structure with little evidence for *α*-helices and *β*-sheets (Avila et al., 2016). Even when bound to tubulin, tau retains an extended structure, with its MTBR domain spanning three tubulin monomers across the longitudinal interface of a protofilament. Tau can remain associated to αβ tubulin dimers over repeated cycles of protofilament assembly and disassembly because it binds in a hydrophobic pocket at the αβ tubulin dimer interface, a region that is structurally unaltered by GTP hydrolysis during microtubule polymerization (Kadavath et al., 2015; Kellogg et al., 2018). Unbound, soluble, monomeric tau adopts a “hairpin” structure in which the N- and C-terminus fold over the MTBR, which is thought to prevent aggregation (Chen D. et al., 2019; Mirbaha et al., 2018).

Post-translational modifications of tau include phosphorylation, acetylation, ubiquitination, methylation, and glycation (Alquezar et al., 2020). These changes contribute to tau function and homeostasis by modulating its catabolism, localization, structure, and interactions with other proteins; they also play a role toward the generation of pathological tau (Binder et al., 2005). Phosphorylation is one of the most common post-translational modifications of tau; it is also one of the most studied, as pathological tau aggregates are known to be hyperphosphorylated. Approximately 20% of tau residues can become phosphorylated (Stoothoff and Johnson, 2005) (Table 1), and the extended structure of tau leaves it prone to such post-translational modification. Most phosphorylation occurs in the PRR or C-terminus immediately downstream of the MTBRs (Kimura et al., 2014). Phosphorylated epitopes can be recognized by several antibodies (Table 2). Tightly regulated phosphorylation and dephosphorylation is thought to control tau-microtubule interactions and is carried out by a repertoire of kinases (Martin et al., 2013). Generally, phosphorylation in or near the MTBR is thought to decrease tau affinity for tubulin due to the addition of a negative phosphate group near the negatively charged MTBRs (Xia et al., 2021). For example, phosphorylated Ser214 and Ser262 have been shown to promote tau detachment from microtubules (Schneider et al., 1999; Sengupta et al., 1998), although these phosphoresidues are also proposed to be neuroprotective, potentially due to a conformation change in the tau protein (Augustinack et al., 2002; Schneider et al., 1999).

**Table 1:** List of potential and reported tau phosphorylation sites in the brain of healthy humans, healthy rhesus macaques, and human Alzheimer’s disease (AD) patients.

Potential phosphorylation sites 2N4R Tau	Healthy human	Healthy rhesus	Human AD
Thr17			
Tyr18	X*		X*
Tyr29			X
Thr30			X
Thr39		X	X
Ser46	X	X	X
Thr50		X	
Thr52			
Ser56		X	X
Ser61		X	
Thr63			
Ser64			
Ser68		X^	X
Thr69		X	X
Thr71		X^	X
Thr76			
Thr95			
Thr101			
Thr102			X
Thr111		X	X
Ser113		X	X
Thr123			
Ser129			
Ser131			
Thr135			
Ser137			
Thr149			
Thr153			X
Thr169			
Thr175		X	X
Thr181	X	X	X
Ser184	X		X
Ser185			X
Ser191		X	X
Ser195			
Tyr197			
Ser198	X	X	X
Ser199	X	X	X
Ser202	X	X	X
Thr205		X	X
Ser208			X
Ser210		X	X
Thr212	X	X	X
Ser214	X	X	X
Thr217	X	X	X
Thr220			X
Thr231		X	X
Ser235	X	X	X
Ser237			X
Ser238		X%	X
Ser241			X
Thr245			
Ser258			X
Ser262	X	X	X
Thr263		X#	X
Ser285			
Ser289			X
Ser293			X
Ser305			X
Tyr310			X
Ser316			X
Thr319			
Ser320			
Ser341			
Ser352			X
Ser356		X	X
Thr361			X
Thr373			
Thr377			
Thr386			X
Tyr394		X	X
Ser396	X	X	X
Ser400	X	X	X
Thr403	X	X	X
Ser404	X	X	X
Ser409		X	X
Ser412	X		X
Ser413	X		X
Thr414	X	X%	X
Ser416	X	X	X
Ser422		X%	X
Thr427			
Ser433			X
Ser435			X

Phosphorylation sites were identified by mass spectrometry in studies of human healthy and AD brains (adapted from Xia et al., 2021) and five rhesus 7–28 years old (Leslie et al., 2021). Residues domains: N-terminus (blue), PRR (orange), MTBR (purple), C-terminus (yellow). X^*^ human site only identified by immunohistochemistry. X^ sites found in a 19-year-old rhesus; X% sites found in a 25-year-old rhesus; X^#^ site found in a 7- and a 15-year-old rhesus.

**Table 2:** List of antibodies and their corresponding target epitopes utilized for histological evaluation of tau and phosphorylated tau reported in the reviewed studies of aged NHPs and NHP models of tauopathy.

Antibody	Epitope
AD1	p-tau; epitope unreported
AD2	p-tau; epitope unreported
Alz50	Misfolded conformation-specific; includes amino acids 2–10 and 312–342
AT8	pSer202/Thr205
AT100	PHF tau at pThr212/pSer214
AT180	pThr231
AT270	pThr181
CP13	pSer202
CP3	pSer214
MC1	Conformation-specific; includes amino acids 7–9 and 312–322
MN423	Tau truncation at Glu391
MV4S4	Normal tau; epitope unreported
M19G	N-terminal tau; epitope unreported
PHF1	PHF tau at pSer396/pSer404
TauC3	Tau truncation at Asp421
Tau2	Non-phosphorylated and phosphorylated tau
Tau5	Amino acids 210–241 of bovine tau; labels human non-phosphorylated and phosphorylated tau; does not label rhesus tau
TG3	PHF conformation-dependent; includes pThr231
TNT2	Amino acids 7–12
TOC1	Oligomeric tau
2B11	Amino acids 301–312
961-S28T	Amino acids 400–427; labels normal and abnormal p-tau

## 3 Tau is highly conserved across primate species

In NHPs, the *MAPT* gene contains the same 16 exons as humans, and the same 8 exons are constitutively expressed. Human and NHP *MAPT* genes are highly homologous in coding regions, which results in conserved 2N4R tau amino acid sequences among species (Figure 1C).

### 3.1 Great apes

All great apes, such as gorillas (genus *Gorilla*), orangutans (genus *Pongo*), chimpanzees and bonobos (both genus *Pan*) express the same six CNS tau isoforms as humans (Holzer et al., 2004). The amino acid sequence of the 2N4R tau isoform in the CNS is 100% identical between humans, chimpanzees, and bonobos (Figure 1C). This isoform is 99.5% identical between humans and gorillas differing at H32L and K87E in the N-terminus. Orangutan and human 2N4R tau are less similar, differing at nine amino acids in the N-terminus. In chimpanzees and gorillas, seven of eight single nucleotide polymorphisms that define the *MAPT* human haplotypes were found to be the H2 sequence (Holzer et al., 2004). Among these two species of great apes, the single nucleotide polymorphisms were conserved and nonpolymorphic (Holzer et al., 2004). A later study demonstrated that both the H1 and H2 haplotypes are present in chimpanzees but probably evolved independently of the human *MAPT* haplotypes. Unlike humans, the H2 haplotype represents the major allele (~56%) of chimpanzees. In contrast, the investigators found that Sumatran orangutans were H2 homozygotes, while a single Bornean orangutan was H1/H2 heterozygous (Zody et al., 2008).

Intronic sequences regulate exon inclusion, and there is evidence that apes have slight differences in noncoding regions from adult humans, which may lead to differences in tau isoform expression. For example, human *MAPT* contains a 59- or 60-bp tandem repeat in intron 9 which controls inclusion of exon 10. Human *MAPT* shares this repeat with gorillas but not always with chimpanzees (Holzer et al., 2004). In an exon trapping experiment, a higher proportion of human and gorilla tau transcripts contained exon 10 than chimpanzees (Holzer et al., 2004). However, on RT-PCR of brain homogenates, chimpanzees and gorillas expressed a higher proportion of exon 10-containing transcripts than humans. Unfortunately, information on tau phosphorylation sites for great apes is not currently available.

### 3.2 Old World monkeys

Old world monkeys, including rhesus (*Macaca mulatta*) and cynomolgus (*Macaca fascicularis*) macaques express the same six CNS tau isoforms as humans. Macaque 2N4R tau is ~98% identical to human 2N4R tau, differing at 9 amino acids in the N-terminal half of the protein. Macaques share their 2N4R tau sequence with baboons (*Papio papio*) and African green monkeys (*Chlorocebus sabaeous*) (Figure 1C). All six tau isoforms are ubiquitously expressed in neurons throughout the rhesus brain (Gambardella et al., 2023). Like great apes, seven of the eight single nucleotide polymorphisms that define the *MAPT* haplotypes were found to have the H2 sequence in macaques (Holzer et al., 2004). Fluorescent *in situ* hybridization on chromosomes from three macaque species (*M. mulatta, fascicularis,* and *arctoides*) demonstrated H2 homozygosity (Zody et al., 2008).

Exon 8, which has not been reported as transcribed in humans, contains an evolutionarily conserved Pro-Pro-Pro motif that introduces a sharp bend into the protein structure. This bent conformation is proposed to be neuroprotective by sterically hindering aggregation. Furthermore, the inclusion of exon 8 introduces a Tyr residue that is proposed to “hinge” the protein at the interface between the N-terminus and MTBR and, thus, modulate its ability to be phosphorylated at Ser202/Thr205. Interestingly, a study in rhesus macaque brains showed that a proportion of tau mRNAs contain exon 8 (Nelson et al., 1996); tau proteins with exon 8 have not yet been reported.

Several 2N4R tau phosphorylation sites were identified in the prefrontal cortex of five rhesus aged 7–28 years by mass spectrometry (Leslie et al., 2021). It is important to note that potential phosphorylation sites between rhesus and human 2N4R tau are highly conserved. Rhesus tau only has three less potential phosphorylation sites than human tau due to evolutionary differences in amino acid sequence (Figure 1C); these are T52A, T95M, and T220A in the N-terminal half. When tau phosphorylation is compared between cortical tissue from rhesus macaques and healthy humans (Leslie et al., 2021; Xia et al., 2021), several similarities and differences emerge (Table 1). First, the MTBR is the most common site of phosphorylation in both species. Second, rhesus tau has more phosphorylated residues on the N-terminal half of the protein. Lastly, rhesus tau had 16 residues that were phosphorylated in all five animals studied, of which seven residues (i.e., Thr39, Thr50, Ser56, Ser61, Thr69, Ser113, and Thr231) were not phosphorylated in healthy humans. Six of these seven residues occur in the N-terminal half, representing the least conserved portion of the protein. Interestingly, Thr231 is phosphorylated in normal rhesus tau (Gambardella et al., 2023), although this site is described as an early marker of tauopathy in human AD (Augustinack et al., 2002). Age-related increases in pSer235-tau and pSer396-tau, but not pThr181-tau, were identified in rhesus 7–28 years old (Leslie et al., 2021).

### 3.3 New World monkeys

Tau studies in New World monkeys have focused on common marmosets (*Callithrix jacchus*) and squirrel monkeys (*Saimiri Sciurus*). The 2N4R tau isoform of both species is missing a 10 amino acid-long sequence in the N-terminal domain, which was previously referred to as the “primate unique motif” (Stefanoska et al., 2018). Thus, it contains only 431 amino acids, opposed to the 441 amino acid-long 2N4R tau present in humans, apes, and Old World monkeys (Figure 1C). Notably, all other primate suborders still retain this sequence. Adult common marmosets were originally reported to exclusively express 4R isoforms in the CNS, like adult rodents (Sharma et al., 2019). Yet, 3R-tau was recently detected in the brains of marmosets aged 1–10 years, albeit to a lesser extent than 4R-tau (Huhe et al., 2024).

Reports on tau phosphorylation sites in New World monkeys are scarce. Tau in the brain of newborn marmosets is highly phosphorylated at several residues, mirroring human fetal tau and tau phosphorylation in disease (e.g., pSer202/Thr205-tau). In comparison, adult marmosets harbor small amounts of pT231-tau, pS396-tau, and pS404-tau (Sharma et al., 2019).

### 3.4 Prosimians

Compared to other NHP species, there is limited information on normal tau in prosimians; reports of tau isoform expression and phosphorylation sites are not available. The 2N4R tau isoform in the gray mouse lemur (*Microcebus murinus*) is 440 amino acids long, with a deletion of a glutamine in the region encoded by exon 7. It has 20 amino acid substitutions in the N-terminal half, as well as a serine addition and lysine deletion in the 1 N insert. There are two amino acid substitutions in the C-terminal half, located in the regions encoded by exons 9 and 13, respectively (Figure 1C).

### 3.5 What can be learned about the development of tau pathologies through the comparison of tau sequences across primate species?

It is well documented that increasing differences in the tau sequence are observed following the evolutionary scale from humans to great apes, then Old World monkeys, New World monkeys, and finally prosimians. If great apes are protected from neurodegenerative disease, this is likely not explained by tau primary sequence, as 2N4R tau is identical in humans, chimps, and bonobos, and 99.5% similar between humans and gorillas. However, the N-terminus of human tau has an increased amount of disorder compared to tau in Old World monkeys, New World monkeys, and prosimians (Trushina et al., 2019). This understanding could provide a basis for further research in novel protein–protein interactions (e.g., tau interaction with synaptic proteins; Stefanoska et al., 2018). Interestingly, the MTBR is conserved between humans and NHPs, including the VQIINK and VQIVYK hexapeptide motifs, which form the core of the protective “hairpin” structure in normal human tau and the core of PHFs in AD. In NHPs, the relationship between tau sequence and conformation has not been studied; this information could be insightful. Furthermore, studies on neuroanatomical tau abundance and tau fragmentation throughout the lifespan have not been conducted in NHPs and could provide insight on potential disease susceptibility.

Regarding tau isoform expression in NHPs and its relationship to disease susceptibility, there are disparities in data suggesting that humans, chimps, and gorillas may express different levels of exon 10-containing transcripts. A repeat in intron 9 may control the inclusion of exon 10 (Holzer et al., 2004). Further, in adult common marmosets, it has been traditionally reported that 3R isoforms are not expressed (Sharma et al., 2019). However, new research challenges this notion (Huhe et al., 2024). These studies allude to a general need for more research on tau isoform expression across the lifespan in NHPs and the corresponding contributions of intronic elements.

Some phosphoresidues that are considered disease markers in humans (e.g., pT231-tau), have been shown to be expressed in healthy young adult macaques (Gambardella et al., 2023). A thorough investigation of normal tau phosphorylation sites identified by mass spectrometry in any NHP species besides rhesus macaques would help determine relevance of disease-associated phosphorylation sites across species. Moreover, studies encompassing the species’ entire lifespan would help understand age-related changes in p-tau expression compared to disease.

## 4 The CNS of asymptomatic aged humans can present sporadic tau pathologies

**Figure 2:**
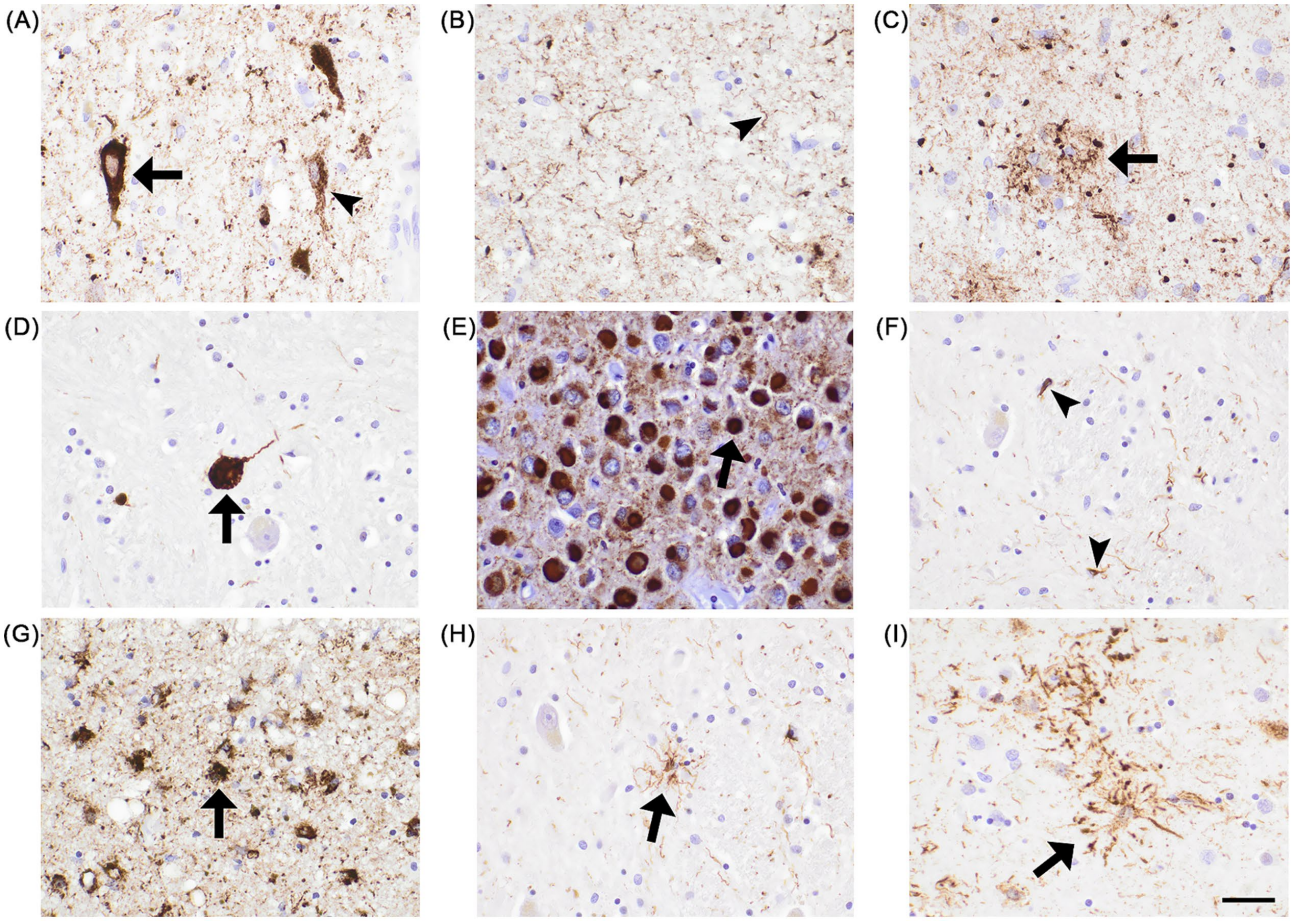
Examples of AT8+ postmortem tau pathologies in human cases of Alzheimer’s disease (AD), progressive supranuclear palsy (PSP), Pick’s disease (PiD), and corticobasal degeneration (CBD). **(A)** Pretangle (arrowhead) and mature NFT (arrow) in the cornu ammonis (AD). **(B)** Dystrophic neurites (arrowhead) in the entorhinal cortex (AD). **(C)** Neuritic plaque (arrow) in the insula (AD). **(D)** Globose NFT (arrow) in the putamen nucleus (PSP). **(E)** Pick bodies (arrow) in the cornu ammonis (PiD). **(F)** Oligodendroglial coiled bodies (arrowheads) in the putamen nucleus (PSP). **(G)** Thorn-shaped astrocyte (arrow) in the subpial medial temporal lobe (AD). **(H)** Tufted astrocyte (arrow) in the putamen (PSP). **(I)** Astrocytic plaque (arrow) in the caudate nucleus (CBD). Scale bar, 100 μm.

Beyond the changes in tau expression across the lifespan mentioned in section 2, the aged human brain may present intracellular aggregates of hyperphosphorylated tau. These inclusions resemble tau pathologies found in tauopathies, of which neurofibrillary tangles (NFTs) are the most widely recognized (Figure 2A). Detection of NFTs typically relies on their positive staining with silver or with the AT8 antibody targeting the pSer202/pThr205 epitopes (Table 2).

Numerous studies of cognitively unimpaired humans (49–105 years old) have reported NFTs in the entorhinal cortex and hippocampus. NFT density is variable between these individuals but is less than that of AD cases (Morris et al., 1991). NFTs have also been reported in brainstem nuclei, the anterior olfactory nucleus, and neocortical areas (Bouras et al., 1993; Nelson et al., 2009; Price et al., 1991), usually to a lesser extent than medial temporal lobe structures.

These publications influenced the establishment of diagnostic criteria for primary age-related tauopathy (PART) (Crary et al., 2014), which refers to elderly subjects that present NFT burden without *β*-amyloid (Aβ) plaques in the brain at autopsy; NFTs must be Braak stage ≤IV (see section 5.2 for explanation on AD staging). Patients with PART are cognitively unimpaired to mild amnestic, with only few patients exhibiting profound impairments. Crary et al. (2014) note that the overwhelming majority of individuals with PART fall into Braak stage I-II, as Braak stage 0 is uncommon in elderly populations, and Braak stage III/IV without Aβ only occurs in 2–10% of PART cases (Kovacs et al., 2013; Nelson et al., 2009; Schneider et al., 2009). The term was coined to facilitate scientific communication, but it is unclear whether PART is a condition associated with normal aging or is an early indicator of AD or other tauopathies.

## 5 Human tauopathies

In this section, we present a summary on human tauopathies as a framework for the analysis of reports on tau pathologies in aged NHPs and NHP models of tauopathies. As mentioned in the introduction, tauopathies are neurodegenerative disorders associated to hyperphosphorylated and aggregated tau inclusions in neurons and/or glia. They are classified as primary or secondary depending on whether accumulation of tau pathologies is the main feature. It is important to consider that patients with the same disease diagnosis can have differing clinical presentations, pathological tau conformations, and/or spatial–temporal tau accumulations (Chung et al., 2021). Patients often present mixed pathologies (i.e., tau with alpha-synuclein or TDP-43), which may contribute to syndrome variation, Conditions related to traumatic brain injury, stroke and environmental toxins can also present tau inclusions (Chen and Jiang, 2019; Edwards et al., 2020), but they are beyond the scope of this review.

In addition to mature NFTs, also termed flame-shaped NFTs (Figure 2A), disease-associated tau inclusions include pretangles (Figure 2B), neuritic plaques (Figure 2C), globose NFTs (Figure 2D), Pick bodies (Figure 2E), coiled bodies (Figure 2F**)**, thorn-shaped astrocytes (Figure 2G), tufted astrocytes (Figure 2H), and astrocytic plaques (Figure 2I). All these tau pathologies are visualized by silver staining or AT8 immunohistochemistry. Currently, clinical tauopathy diagnosis is ultimately confirmed by neuropathological evaluation at autopsy.

### 5.1 Primary tauopathies

All primary tauopathies present pathological tau aggregation, yet they differ on the predominant tau isoform accumulated, affected brain regions, and affected cell type (Chung et al., 2021).

FTD associated with tau accumulation (FTD-tau) is considered a typical primary tauopathy. FTD is the most common form of dementia in patients under 60 years old. It is an umbrella term for disorders presenting with frontal and/or temporal lobe atrophy associated to abnormal accumulation of proteins, such as tau, TDP-43, or FUS (Bang et al., 2015). FTD-tau can be genetic or sporadic; clinical presentations are diverse but fall into three main categories: behavioral variant FTD, progressive primary aphasia, or FTD with amyotrophic lateral sclerosis or parkinsonism (Siuda et al., 2014; Waldö, 2015). Outside of these classifications, progressive cognitive impairment can also be a presenting symptom (Beber and Chaves, 2013). Over 50 mutations in *MAPT* have been linked to genetic FTD-tau, including *MAPT* P301S, S320F, and R406W (for review see Strang et al., 2019). Genetic FTD linked to *MAPT* mutations are denoted FTD associated to chromosome 17 (FTD-17), sometimes termed FTDP-17 if parkinsonism is clinically present (Boeve and Hutton, 2008). Progression and distribution of tau pathologies in FTD varies between FTD subtype and clinical presentation but typically does not follow an AD pattern (Reed et al., 1997). Due to the diversity of neuropathological burden in FTD syndromes, a unified staging system is not possible. Interestingly, an unexpected link between FTD and cerebellar atrophy has recently been discovered (Bocchetta et al., 2016; Bussy et al., 2023; Chen Y. et al., 2019); more research is needed to understand the contribution of different *MAPT* mutations or sporadic tau pathologies to cerebellar atrophy.

Subtypes of FTD-tau include Pick’s disease (PiD), progressive supranuclear palsy (PSP), and corticobasal degeneration (CBD); they can be sporadic or genetic. An increased risk for sporadic PiD has been linked to tau haplotype H2; rare mutations or duplications in *MAPT* have been linked to genetic PiD (Valentino et al., 2024). For PSP, *MAPT* is the strongest risk locus, with 15 *MAPT* mutations to date associated with the disease (Wen et al., 2021). With respect to CBD, *MAPT* mutations are the second most common, after *GRN* (Arienti et al., 2021). The differential diagnosis between these three diseases is based on clinical presentation and ultimately postmortem neuropathological findings. The pathological hallmark of PiD is the presence of cytoplasmic, round, and exclusively 3R-tau+ inclusions called Pick bodies (Figure 2E). Tau neuropathology in PiD is proposed to originate in the limbic/paralimbic cortices and progress through subcortical regions (Irwin et al., 2016). Neuronal loss predominates in the frontal neocortex, followed by the temporal cortex, parietal cortex, hippocampus, and finally the amygdala (Kovacs, 2015). PSP and CBD are characterized by only 4R-tau+ inclusions. In PSP, globose NFTs (Figure 2D) are found in the basal ganglia, diencephalon, and brainstem nuclei and neuronal loss predominates in the subthalamic nucleus and substantia nigra. Tufted astrocytes (Figure 2H) are found in the precentral gyrus, striatum, and superior colliculus, and occasionally in the thalamus, subthalamic nucleus, red nucleus, and cortex. Oligodendroglial coiled bodies (Figure 2F) are found throughout the white matter. It should be noted that the PSP clinical subtypes have specific patterns of progressive tau accumulation and associated neuronal degeneration and that the non-neuronal cell types affected depend on clinical presentation (Kovacs et al., 2020). In CBD, globose NFTs (Figure 2D) are seen in the substantia nigra and locus coeruleus. Atrophy occurs in the frontal and/or parietal regions with the presence of ballooned neurons; the subthalamic nucleus is typically spared. Astrocytic plaques (Figure 2I) are characteristic of CBD, mainly in the neocortex and striatum. Oligodendroglial coiled bodies (Figure 2F) are also present in the striatum, pallidum, and thalamic fascicle, but usually to a lesser extent than in PSP (Kovacs, 2015; Spina et al., 2019). Importantly, tau phosphorylation at Ser422 followed by truncation at Asp421 were found to be precursors to neuronal tau inclusions such as NFTs and Pick bodies, but not the glial inclusions found in PSP and CBD (Guillozet-Bongaarts et al., 2007).

Argyrophilic grain disease (AGD) is a late-onset dementia, sometimes classified as a form of FTD-tau. PSP is the most frequent clinical diagnosis for patients with AGD pathology, yet clinical manifestations also include amnestic cognitive impairment that is typically milder than that of AD, or less commonly behavioral disturbances (Gil et al., 2018; Steuerwald et al., 2007). AGD has been linked to higher frequency of the *MAPT* H1/H1 haplotype (Gil et al., 2018). Like PSP and CBD, AGD is a 4R-tauopathy. It is characterized by small, argyrophilic, dot or comma-shaped inclusions in neuronal dendrites known as argyrophilic grains; this pathology is found mainly in the entorhinal cortex and hippocampus. Other pathologies include pretangles (Figure 2A) in limbic projection neurons and oligodendroglial coiled bodies (Figure 2F) in the hippocampal and peri-amygdaloid white matter (Ferrer et al., 2008). Accumulation of tau pathology in AGD is proposed to start in the ambient gyrus, extending to the anterior and posterior medial temporal lobe, and finally the septum, insular cortex and anterior cingulate gyrus (Saito et al., 2004). Although severe atrophy of the affected regions is typically observed, cognitive impairment tends to be milder than other types of dementia. Ballooned neurons in the amygdala are evident (Tolnay and Probst, 1998).

### 5.2 AD is a secondary tauopathy

AD is the leading cause of dementia in patients over 65 years of age. Most AD cases are sporadic; only 5% are genetic. Mutations in *APP, PSEN1,* and *PSEN2* are known to cause autosomal dominant AD; all of them increase amyloid burden by affecting processing of the amyloid precursor protein. In addition, over 70 genes or loci have been proposed to contribute to AD risk (Reitz et al., 2023), such as the *APOE*-*ε4* allele. Although tauopathies are a predominant feature of AD, a causative link between *MAPT* mutations and AD has not been identified. Moreover, the underlying mechanism linking Aβ and tau remains elusive.

In AD, 3R-tau+ and 4R-tau+ intraneuronal inclusions follow the accumulation of extracellular Aβ plaques. These include classic flame-shaped NFTs (Figure 2A), neuropil threads (NTs), and plaque-associated tau+ dystrophic neurites (Figures 2B,C). Tau pathologies first appear in the entorhinal cortex, then spread topographically to the hippocampus, limbic structures, and finally the neocortex (Braak and Braak, 1991). In AD, tau undergoes sequential phosphorylation and conformational changes that are thought to trigger the formation of paired helical filaments (PHFs), the main tau conformation within NFTs (Binder et al., 2005; García-Sierra et al., 2003; Mondragón-Rodríguez et al., 2008). P-tau epitopes, such as T181 and T231, may represent early stages of pathology, whereas pS202/T205 and pS396/404 represent late stage of pathology (Augustinack et al., 2002). Two hexapeptide motifs in the MTBR, VQIINK and VQIVYK, form the core of AD PHFs; these motifs are shielded from the extracellular environment in native tau but are exposed in tau monomers isolated from AD brains (Chen et al., 2019).

AD diagnosis is confirmed postmortem by the presence of both Aβ plaques and AT8+ or silver+ NFTs. As mentioned above, Aβ plaque severity is rated according to Thal staging (Thal et al., 2002), while the severity of neurofibrillary changes (NFTs, NTs, and pretangles) are rated according to Braak staging (Braak and Braak, 1991). Although many tau antibodies (Table 2), recognize disease-associated tau phosphoresidues and/or aberrant tau conformations, AT8-immunoreactivity (-ir) is used as the standard for Braak staging (Del Tredici and Braak, 2020). In this system, stages I-II correspond to NFT accumulation in the transentorhinal regions, II-III adds presence of NFTs in the limbic regions, and V-VI are considered end-stages of AD with widespread NFTs in the isocortical association areas.

## 6 Aged NHPs can present sporadic tau pathologies in the CNS

**Figure 3:**
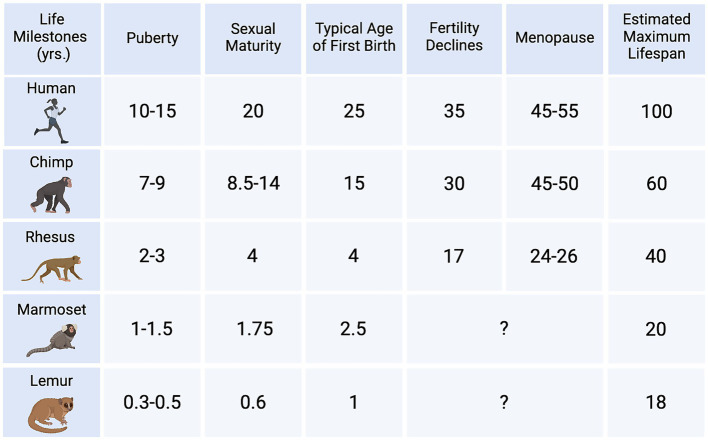
Approximate age in years (yrs.) of life milestones across female species from representative primate families. Chimpanzees reach sexual maturity between 8.5–14 yrs. of age and are on average 15 yrs. old when they first give birth (Walker et al., 2018); their fertility declines in their 30s and experience menopause at 45–50 yrs. old (Wood et al., 2023). In rhesus, first birth often occurs at age 4 (Pittet et al., 2017) and their fertility declines around age 17 (Lee et al., 2021); menopause was reported by 24–26 yrs. old (Walker, 1995). Common marmosets are considered primed adults between 2 and 8 yrs. of age, although they do not seem to undergo reproductive senescence (Abbott et al., 2003). Gray mouse lemurs reach sexual maturity around 0.5 yrs. (Hohenbrink et al., 2015) and, like common marmosets, do not seem to experience reproductive senescence. Their median lifespan is 5.5 yrs. (Martine and Aude, 2022) but can live up to 18 yrs. in captivity. Listed maximum lifespans in NHPs are estimated for captive animals. Mature tau pathologies, like NFTs, have been reported in extremely old animals nearing their respective species’ maximum lifespan, yet without behavioral or clinical correlates, it is difficult to assert whether tau pathology affects survivability. Created in https://BioRender.com.

To delve into the question of whether NHPs present age-related tau pathologies we search in PubMed up to December 2024. Keywords included “nonhuman primate,” “monkey,” “marmoset,” “macaques,” “prosimian,” “ape,” “tau,” “neurofibrillary tangles,” “tauopathies,” “phosphorylated tau,” “age,” “old” combined with peer-reviewed and English language as filters. Reference lists in original research and review articles were examined to gather additional publications. Title and abstracts were screened for topic relevance. Fifty-two articles describing original peer-reviewed studies in NHPs were selected and analyzed to extract information on findings per species; see Tables 3–6 for compiled references and corresponding data. To understand the frequency of tau pathologies in NHPs, we considered the number of studies and subjects, as well as the number of positive cases detected and their age; see Figure 3 for a comparison of ages across human and example NHP species based on biological milestones.

**Table 3:** Peer-reviewed publications on tau immunoreactivities in aged great apes.

References	Species	*n* Total	Age (yrs.)	n aged ≥30 yrs./nTotal	Silver+ or ThioS+ NFTs (n+/nTotal)	AT8+ NFTs (n+/nTotal)	AT8-ir (n+/nTotal)	Other tau antibodies (n+/nTotal)	Notes
Gearing et al. (1994)	Chimpanzee (*P. troglodytes*)	3	45, 56, 59	3/3	0/3	NE	NE	Alz50: 3/3; rare neurons in lateral Pu PHF1, Tau-1: 0/3	**-**
Kuroki et al. (1997)	Chimpanzee (*P. troglodytes*)	1	38	1/1	0/1	NE	NE	Tau2: 1/1; neurons and glia in SN, RN, DN, hpc, cerebellar Purkinje cells	**-**
Rosen et al. (2008)	Chimpanzee (*P. troglodytes*)	1	41	1/1	1/1	1/1	1/1; neurons, NTs, and NCs mostly in neoctx, occasionally hpc	CP13: 1/1; neoctx, hpc PHF1, MC1: 1/1; neoctx	NFTs reported as CP13+, PHF1+, MC1+; images were not provided
Rosen et al. (2011)	Chimpanzee (*P. troglodytes*)	3	41, 44, 47	3/3	NE	1/3; same NHP as Rosen et al. (2008)	1/3; same NHP as Rosen et al. (2008)	CP13, PHF1, MC1: 1/3*	*Authors report “little to no aberrant tauopathy in the NHPs, with the exception of one aged chimpanzee as previously described (Rosen et al., 2008)”
Edler et al. (2017)	Chimpanzee (*P. troglodytes*)	20	37–62	20/20	5/20; 45, 49, and 58 yrs. Braak I/II, 57 yrs. Braak V, 39 yrs. NFTs mainly in pfc	5/20; 45, 49, and 58 yrs. Braak I/II, 57 yrs. Braak V, 39 yrs. NFTs mainly in pfc	20/20; pretangles in neoctx and/or hpc 12/20; 37-58 yrs., dystrophic neurites in neoctx and/or hpc	NE	**–**
Rogers Flattery et al. (2020)	Chimpanzee. (*P. troglodytes*)	6	29–43	5/6	NE	NE	NE	CP13: 6/6; variable accumulation in neurites and few soma in temporal lobe	**–**
Kimura et al. (2001)	Gorilla, western lowland (*G. gorilla gorilla*)	1	44	1/1	0/1	NE	NE	Tau2: 0/1	**–**
Perez et al. (2013)	Gorilla, western lowland (*G. gorilla gorilla*)	9	13–55	7/9	0/9	0/9	1/9*; astrocytes, neurons, and NCs in neoctx	Alz50: 9/9; scattered and fusiform soma and processes in neoctx and hpc, few NCs in neoctx MC1: 3/9**; NCs in neoctx, glia-like CBs at interface of neoctx GM and WM	*AT8 reported in a 55 yrs., unclear if more NHPs were AT8+ **MC1+ images shown from 49 yrs., authors report MC1-ir in the “oldest gorillas” which may also include 50 and 55 yrs.
Perez et al. (2016)	Gorilla, wild mountain (*G. beringei beringei*)	10	16–42 (estimated)	7/10	0/10	0/10	8/10; glia and NCs in frontal ctx	Alz50: 8/10; glia and NCs in frontal ctx	**-**
Selkoe et al. (1987)	Orangutan (*P. pygmaeus*)	1	46	1/1	0/1*	NE	NE	Antisera raised against human PHFs: 0/1	*ThioS staining only.
Gearing et al. (1997)	Orangutan (scientific name NR)	4	10, 28, 31, 36	2/4	0/4	NE	NE	Tau (epitope NR): 0/4	-

Results are reported as number of positive NHPs to total number of NHPs in the study (n+/nTotal); age, brain region and morphological description are included if available. *Details specific issues in the publication of the corresponding row denoted with corresponding asterisk(s). CBs, coiled bodies; ctx, cortex; DN, dentate nucleus; GM, gray matter; hpc, hippocampus; −ir, immunoreactivity; NC, neurite clusters; NE, not evaluated; neoctx, neocortex; NFT, neurofibrillary tangle; NHPs, nonhuman primates; NR, not reported; NT, neuropil threads; pfc, prefrontal cortex; PHFs, paired helical filaments (differentiated from the antibody PHF1); Pu, putamen; RN, red nucleus; SN, substantia nigra; ThioS+, Thioflavin S positive; WM, white matter; yrs., years.

**Table 4:** Peer-reviewed publications on tau immunoreactivities in aged Old World monkeys.

References	Species	*n* Total	Age (yrs.)	n aged ≥23 yrs./nTotal	Silver+ or ThioS+ NFTs (n+/nTotal)	AT8+ NFTs (n+/nTotal)	AT8-ir (n+/nTotal)	Other tau antibodies (n+/nTotal)	Notes
Struble et al. (1985)	Rhesus (*M. mulatta*)	15	4–31	unclear	0/15	NE	NE	NE	–
Selkoe et al. (1987)	Rhesus (*M. mulatta*)	3	30–31	3/3	0/3	NE	NE	Antisera raised against human PHFs: 0/3	–
Gearing et al. (1994)	Rhesus (*M. mulatta*)	4	20, 21, 29, 30	2/4	0/4	NE	NE	PHF1: 0/4	–
Härtig et al. (2000)	Rhesus (*M. mulatta*)	1	28	1/1	0/1	0/1	1/1; neuronal soma and processes in ec and hpc	AT100, PHF1, TG3: 1/1; few neurons in hpc and ec	–
Shah et al. (2010)	Rhesus (*M. mulatta*)	11	25–31	11/11	0/11	NE	NE	PHF1: 11/11; neocortical NCs	–
Rosen et al. (2011)	Rhesus (*M. mulatta*)	9	20–38	8/9	NE	?*	?*	CP13, PHF1, MC1:? *	*Authors report “little to no aberrant tauopathy in the NHPs”
Carlyle et al. (2014)	Rhesus (*M. mulatta*)	8	9–31	5/8	NE	NE	NE	pS214-tau: 8/8*; neuronal synapses in the dlPFC	*Presumed to be all NHPs, n positive NHPs NR
Paspalas et al. (2018)	Rhesus (*M. mulatta*)	10*	7–38	8/10	NE	1/10; 38 yrs., layer II/V ec	8/10; >24 yrs., layer II stellate cells in ec 3/10; both pretangles and healthy pyramidal cells in deeper ec layers 1/10; 33 yrs., sporadically in dlPFC	CP3: 2/10**; 7–9 yrs., neurons in ec, and 3/10**; 31-34 yrs., neuronal synapses in dlPFC AT180: 2/10***; NTs in ec AT270: 2/10***; neuronal soma and dendrites in ec	*10 NHPs used for IHC **CP3-ir results NR in 5 NHPs aged 24, 26 (*n* = 2), 28, and 38 yrs. ***AT180-ir and AT270-ir results shown in 2 NHPs aged 33 and 34 yrs.
Zhou et al. (2018)	Rhesus (*M. mulatta*)	8	22–34	6/8	NE	NE	NE	PHF1: 0/3 pS199/202-tau: 0/3	-
Crimins et al. (2019)	Rhesus (*M. mulatta*)	16	9–26	8/16	NE	NE	NE	CP3: 16/16*; neuronal synapses in the dlPFC	*Presumed to be all NHPs, n positive NHPs NR
Zhang et al. (2019)	Rhesus (*M. mulatta*)	16	5–31	unclear (*n* = 8, 19-31 yrs.)	0/5*	0/5*	0/5*	NE	*Only 5 NHPs were Aβ+ and subsequently stained for AT8
Datta et al. (2021)	Rhesus (*M. mulatta*)	9	7–30	5/9	0/9	0/9	1/9*; 30 yrs., neuronal soma and dendrites in dlPFC	pT217-tau: 1/9*; 30 yrs., neuronal soma and dendrites in dlPFC pS214-tau: 1/9*; 26 yrs., dlPFC pyramidal cell soma and dendrites, and diffuse in neuropil	*Data only shown from 1 NHP, n positive NHPs NR
Barnes et al. (2024)	Rhesus (*M. mulatta*)	31*	7–33	16/31	0/31	0/31	11/30**; ≥24 yrs. and a single 16 yrs., pretangles, occasional tufted astrocytes and oligodendroglial CBs, Braak I-III, densities greatest in amg, then septum, hpc/ transentorhinal, hyp, frontal ctx, least in cg/temporal ctx	NE	*31 NHPs used for IHC **30 NHPs were reported in Table 5 of Barnes et al. (2024)
Podlisny et al. (1991)	Cynomolgus (*M. fascicularis*)	3	19	0/3	0/3	NE	NE	Antisera raised against human PHFs: 0/3	-
Kiatipattanasakul et al. (2000)	Cynomolgus (*M. fascicularis*)	1	>35*	1/1	1/1, thl	NE	NE	Argyrophilic and weakly Tau2 + GFTs in striatum, thl, SN, RN, GP, trapezoid body, pyramid, pons, and MO, less common in cerebral/cerebellar WM, crus cerebri, and hpc	*Specific age NR
Kimura et al. (2003)	Cynomolgus (*M. fascicularis*)	28*	4–36	5/30	0/28*	0/28*	0/28*	NE	*28 NHPs used for IHC
Oikawa et al. (2010)	Cynomolgus (*M. fascicularis*)	7*	6–36	5/7	2/7; 32 and 36 yrs., temporal ctx	2/7; 32 and 36 yrs., temporal ctx	4/7; ≥26 yrs., temporal ctx glia, neurons, NCs, and hpc glia and neurites	NE	*7 NHPs used for IHC
Darusman et al. (2014)	Cynomolgus (*M. fascicularis*)	8	7–30	6/8	NE	NE	NE	pT231-tau: 1/8; 29 yrs., neuronal cytoplasm in temporal and occipital lobes	-
Okabayashi et al. (2015)	Cynomolgus (*M. fascicularis*)	22	6–28	10/22	NE	0/22	0/22	NE	NHPs were normal controls for a study on diabetes and AD
Uchihara et al. (2016)	Cynomolgus (*M. fascicularis*)	20	7–36	12/20	1/20; 36 yrs., scarce NFTs in hpc pyramidal layer	1/20; 36 yrs., scarce NFTs in hpc pyramidal layer	5/20; pretangles and astrocytes in neoctx and hpc pyramidal neurons, fiber bundles and oligodendroglia-like cells in WM of GP and hpc	AT8 colocalized with 4R-tau but not 3R-tau	-
Zhou et al. (2018)	Cynomolgus (*M. fascicularis*)	3	29, 30.5, 32	3/3	NE	NE	NE	PHF1: 0/3 pSer199/202-tau: 0/3	-
Jester et al. (2022)	Cynomolgus (*M. fascicularis*)	6	14–19	0/6	0/6	0/6	6/6; neuronal soma and processes in pfc and hpc	pT231-tau: 6/6; neuronal soma and processes in pfc and hpc	-
Kuroki et al. (1997)	Japanese (*M. fuscata*)	2	28, 41	2/2	0/2	NE	NE	Tau2: 2/2	-
Schultz et al. (2000a)	Baboon (*P. hamadryas*)	4	20, 24, 26, 30 (estimated)	3/4	2/4*; 26 and 30 yrs., hpc	2/4*; 26 and 30 yrs.	2/4*; 26 and 30 yrs., neurons and glia in hpc, amg	AT100, AT270, Alz50: listed, results NR PHF1, TG3: 1/4*; astrocytes in hpc and oligodendroglial CBs in perforant path 92e: 1/1**; filaments in oligodendroglial CBs, astrocytes, and granule cells in the fascia dentata	*Authors mention single tau+ inclusions in the 2 younger NHPs, antibody used NR **Images only from the 30 yrs. old ***Only 30 yrs. old stained for 92e
Schultz et al. (2000b)	Baboon (*P. anubis and P. hamadryas*)	50	1–30	25/50	1/50; 30 yrs. from *hamadryas* subgenus	1/50; 30 yrs. from *hamadryas* subgenus	26/50; ≥19 yrs., hpc pyramidal neurons, fascia dentata granule cells, and layer II ec, oligodendroglial CBs in entorhinal perforant path, fimbria, and fornix, astroglia in hpc, subgranular plexiform layer, subependymal regions of lateral and third ventricles, subpial periamygdaloid ctx and basal mamillary region	PHF1, TG3: 26/50*	4 *P. hamadryas* were from (Schultz, Dehghani, et al., 2000) *Authors report “Both the neuronal and glial cytoskeletal changes in baboons are labeled by AT8, PHF1, and TG3”
Härtig et al. (2000)	Baboon (*P. hamadryas*)	2	26, 28	2/2	0/2	0/2	2/2; rare neurons and glia in frontal ctx	NE	–
Ndung'u et al. (2012)	Baboon (*P. hamadryas, cynocepahlus and anubis*)	6	18–27	3/6	0/6	0/6	3/6; few neurons in temporal ctx	NE	–
Lemere et al. (2004)	African green monkey (*C. aethiops*)	3	15, 22, 30	1/3	0/3	0/3	1/3; 30 yrs., NCs in temporal ctx	NE	–
Cramer et al. (2018)	African green monkey (*C. aethiops*)	15*	7–32	5/15*	2/15; >20 yrs., hpc and ec	2/15; >20 yrs., hpc and ec	2/15; >20 yrs., in NFTs in hpc and ec	NE	*15 NHPs used for IHC
Latimer et al. (2019)	African green monkey (*C. aethiops sabaeus*)	18	8–23	4/18	0/18	0/18	18/18; granular cytoplasmic -ir in small cells throughout neoctx, mainly superior occipital gyrus, also striatum and GP, least common in hpc	NE	–
Corey et al. (2023)	African green monkey (*C. sabaeus*)	31*	3–25	unclear (*n* = 11; >15 yrs.)	NE	0/31	0/31	CP13**: 14/31 total; 3-25 yrs.; neurons, neuropil, occasional oligodendroglial-like cells; 10/10, cg; 9/10, orbital ctx; 8/10, pfc; 7/12, ec; 6/12, sub; 12/12, hpc CA1/CA3; 3/12, hpc DG	*31 NHPs used for IHC **As reported by authors in Table 2 ofCorey et al. (2023)
Härtig et al. (2000)	Campbell’s guenon (*C. mona campbelli*)	2	27, 30	2/2	0/2	0/2	2/2; sparse cells in cg	NE	–

Results are reported as number of positive NHPs to total number of NHPs in the study (n+/nTotal); age, brain region and morphological description are included if available. *Details specific issues in the publication of the corresponding row denoted with corresponding asterisk(s). Aβ, amyloid beta; amg, amygdala; CA, cornu ammonis; CBs, coiled bodies; cg, cingulate cortex; ctx, cortex; DG, dentate gyrus dlPFC, dorsolateral prefrontal cortex; ec, entorhinal cortex; GFTs, glial fibrillary tangles; GP, globus pallidus; hpc, hippocampus; hyp, hypothalamus; IHC, immunohistochemistry; −ir, immunoreactivity; MO, medulla oblongata; NC, neurite clusters; NE, not evaluated; neoctx, neocortex; NFT, neurofibrillary tangle; NHPs, nonhuman primates; NR, not reported; NT, neuropil threads; pfc, prefrontal cortex; PHFs, paired helical filaments (differentiated from the antibody PHF1); RN, red nucleus; SN, substantia nigra; sub, subiculum; ThioS+, Thioflavin S positive; thl, thalamus; WM, white matter; yr(s)., year(s).

**Table 5:** Peer-reviewed publications on tau immunoreactivities in aged New World monkeys.

References	Species	*n* Total	Age (yrs.)	n aged ≥8 yrs./nTotal	Silver+ or Thios+ NFTs (n+/nTotal)	AT8+ NFTs (n+/nTotal)	AT8-ir (n+/nTotal)	Other tau antibodies (n+/nTotal)	Notes
Geula et al. (2002)	Common marmoset (*C. jacchus*)	22	2–15	11/22	NE	NE	NE	PHF1, pS262-tau: 0/22	**-**
Rodriguez-Callejas et al. (2016)	Common marmoset (*C. jacchus*)	11	1–18*	7/11**	NE	NE	NE	pT231-tau, AT100: 11/11***; cytoplasm in hpc, ec, temporal ctx, pT231-tau: 2/11; neuronal soma and dendrites in hpc, ec, temporal and parietal ctx Alz50: 11/11; neuronal and possible glial cytoplasm in hpc, ec, temporal and parietal ctx, 2/11; neuronal dendrites and glia-like cells hpc, ec, temporal and parietal ctx	*Exact ages NR. Four groups: adolescent (mean 1.6 yrs.), adult (mean 5.5 yrs.), old (mean 11 yrs.), aged (mean 18 yrs.) ** n unclear, 7 NHPs between “old” and “aged” groups ***Presumed to be all NHPs, n positive NHPs NR
Rodríguez-Callejas et al. (2023)	Common marmoset (*C. jacchus*)	15	1–19	8/15	NE	NE	NE	AT100*: 15/15; astrocytes in hpc and ec	*Presumed to be all NHPs, n positive NHPs NR
Freire-Cobo et al. (2023)	Common marmoset (*C. jacchus*)	17	7–10	unclear	NE	0/17	0/17	NE	**-**
Lemere et al. (2008)	Cotton top tamarin (*S. oedipus*)	36	6–21	29/36	0/36	0/36	0/36	NE	**-**
Selkoe et al. (1987)	Squirrel monkey (*S. sciureus*)	2	20, 23	2/2	NE	NE	NE	Antisera raised against purified PHFs from human AD cerebral ctx: 0/2	**-**
Walker et al. (1987)	Squirrel monkey (*S. sciureus*)	8	8–23*	8/8	0/8	NE	NE	NE	*Estimated to be 8 yrs. (*n* = 3), 12 yrs. (*n* = 1), and 23 yrs. (*n* = 4)
Elfenbein et al. (2007)	Squirrel monkey (*S.* spp.)	15	7, 15–24	14/15	0/15	0/15	NR/15*; occasional cortical neurons and plaque-associated neurites	Alz50: NR/15*; occasional cortical neurons and plaque-associated neurites	*Images not shown
Rosen et al. (2011)	Squirrel monkey (*S. sciureus*)	6	15–23	6/6	NE	NR*	NR*	CP13, PHF1, MC1: NR*	*Authors report “little to no aberrant tauopathy in the NHPs”
Rosen et al. (2016)	Squirrel monkey (*S. sciureus*)	7	14–23	7/7	NE	NR	NR	CP13: 1/7*; rare cortical neurons and neurites PHF1: listed, results NR MC1: 0/7	*A single image of a CP13 + cortical neuron is shown, n positive NHPs NR

Results are reported as the number of positive NHPs to total number of NHPs in the study (n+/nTotal); age, brain region and morphological description are included if available. *Details specific issues in the publication of the corresponding row denoted with corresponding asterisk(s). ctx, cortex; ec, entorhinal cortex; hpc, hippocampus; −ir, immunoreactivity; NE, not evaluated; NFT, neurofibrillary tangle; NHPs, nonhuman primates; NR, not reported; PHFs, paired helical filaments (differentiated from the antibody PHF1); ThioS+, Thioflavin S positive; yrs., years.

**Table 6:** Peer-reviewed publications on tau immunoreactivities in aged prosimians.

References	Species	*n* Total	Age (yrs.)	n aged ≥5 yrs./nTotal	Silver+ or ThioS+ NFTs (n+/nTotal)	AT8+ NFTs (n+/nTotal)	AT8-ir (n+/nTotal)	Other tau antibodies (n+/nTotal)	Notes
Bons et al. (1992)	Gray mouse lemur (*M. murinus*)	25	2–11*	10/25	0/25	NE	NE	NE	*Two groups: young (2-3 yrs.) and aged (8-11 yrs.)
Bons et al. (1995a, 1995b)	Gray mouse lemur (*M. murinus*)	27	1–13	23/27	NE	NE	NE	MV4S4: 22/27; thin granulations in cortical pyramidal neurons 961-S28T: 26/27; neurons in frontal and occipital lobes PHF-tau: 9/27; pyramidal neurons in fronto-parietal and occasionally occipital ctx	**-**
Bons et al. (1995a, 1995b)	Gray mouse lemur (*M. murinus*)	35	2–13	30/35	NE	NE	NE	MV4S4: 28/35; thin granulations in cortical pyramidal neurons, perikarya, and neurites 961-S28T: 34/35; thick granules at periphery of cell bodies and scattered broken neurites in parenchyma PHF-tau, AD2: 15/35; neuronal perikarya AD1, M19G: listed, results NR	**-**
Giannakopoulos et al. (1997)	Gray mouse lemur (*M. murinus*)	40	1–13	30/40	NE	NE	NE	961-S28T: 40/40; layers IV-VI frontal, parietal, and temporal ctx, layers III-V occipital ctx, and subiculum	**-**
Kraska et al. (2011)	Gray mouse lemur (*M. murinus*)	5	5.5–11.3	5/5	NE	NE	NE	CP13: 3/5; faint -ir in few hpc cells	**-**
Schmidtke et al. (2020)	Gray mouse lemur (*M. murinus*)	31	5–13.5	31/31	NE	NE	NE	CP13, PHF1: 0/31	**-**
Härtig et al. (2000)	Brown lemur (*L. fulvus mayottensis*)	2	12, 14	2/2	NE	0/2	1/2; brain region and cell morphology NR	NE	**-**

Results are reported as number of positive NHPs to total number of NHPs in the study (n+/nTotal); age, brain region and morphological description are included if available. *Details specific issues in the publication of the corresponding row denoted with corresponding asterisk(s). ctx, cortex; hpc, hippocampus; −ir, immunoreactivity; MO, medulla oblongata; NC, neurite clusters; NE, not evaluated; neoctx, neocortex; NFT, neurofibrillary tangle; NR, not reported; PHF, paired helical filament (differentiated from the antibody PHF1); ThioS+, Thioflavin S positive; yrs., years.

Our systematic analyses of the literature identified a few reports of sporadic tau inclusions in the brain of aged NHPs. Our criteria for detection of NFTs (or other disease-associated tauopathies) in a publication was the authors’ description of the intraneuronal inclusion positive for silver and/or AT8 staining, with a corresponding image. We differentiated between AT8+ inclusions from AT8-ir and included descriptions of tau immunoreactivities detected by other tau antibodies. Most studies of age-related tau pathologies were performed in Old World monkeys. Captive animals were examined, except for one study in wild mountain gorillas; behavioral evaluations were seldom performed. Compared to human studies, which utilized silver and/or AT8 staining for confirmation of tau pathology, especially for NFTs, NHP studies have utilized a variety of tau antibodies.

### 6.1 Aged great apes

Eleven studies have examined great apes for evidence of tau pathologies encompassing 34 chimpanzees, 20 gorillas, and 5 orangutans; most of the animals were >30 years old (Table 3). Overall, NFTs were reported in six chimps aged ≥39 years (Edler et al., 2017; Rosen et al., 2008). Rosen et al. (2008) identified a 41-year-old chimp that had NFTs highly similar to those found in human disease. Edler et al. (2017) reported NFTs in five chimps of which three were classified with Braak staging 1/II and one with stage V; the fifth chimp had NFTs exclusively in the prefrontal cortex. NFTs have not been identified in gorillas and orangutans.

With regards to AT8-ir, chimps aged ≥37 years presented AT8+ pretangles, neurite clusters, and NTs mainly in the neocortex (Edler et al., 2017; Rosen et al., 2008). In gorillas, AT8+ pretangles were not observed, although AT8+ glia and neurite clusters were found in the frontal cortex of eight ≥35-year-old wild mountain gorillas and a 55-year-old Western lowland gorilla (Perez et al., 2013; Perez et al., 2016). The latter 55-year-old gorilla also had AT8+ glia and neurite clusters in the hippocampus.

Other tau antibodies have been utilized to identify tau accumulations in great ape brains. Notably, a 41-year-old chimp had PHF1-ir and MC1-ir in neuritic plaques and threads, but to a lesser extent than AT8-ir (Rosen et al., 2008). Aged Western lowland gorillas (≥49 years) had occasional Alz50-ir and MC1-ir neurite clusters in the neocortex (Perez et al., 2013). Wild mountain gorillas aged ≥16 years had rare Alz50-ir NCs in the frontal cortex (Perez et al., 2016).

### 6.2 Aged Old World monkeys

In comparison to great apes, 32 reports in Old World monkeys studied the presence of tau pathologies in 141 rhesus macaques, 100 cynomolgus macaques, 2 Japanese macaques, 62 baboons, 67 African green monkeys, and 2 Campbell’s guenon, with ages ranging 1–41 years (Table 4). NFTs were reported by Paspalas et al. (2018) in the entorhinal cortex of one 38-year-old rhesus monkey, while Kiatipattanasakul et al. (2000), Oikawa et al. (2010), and Uchihara et al. (2016) found them in the thalamus, entorhinal cortex, or hippocampus of four cynomolgus macaques aged ≥32 years. Schultz et al. (Schultz, Dehghani, et al., 2000) described NFTs in the hippocampus of two ≥26-year-old baboons and Cramer et al. (2018) in the entorhinal cortex and hippocampus of two African green monkeys aged ≥20 years. NFTs were not detected in aged Japanese macaques and Campbell’s guenons (Härtig et al., 2000; Kuroki et al., 1997).

AT8-ir has been detected in the brain of aged Old World monkeys. Six studies reported results of AT8 immunostaining in rhesus. In these animals, neuronal AT8-ir was found in the entorhinal cortex and hippocampus starting around ≥24 years of age, although one 16-year-old monkey harbored pretangles restricted to the amygdala (Barnes et al., 2024). Four ≥28-year-old rhesus from three separate studies had amygdala and/or prefrontal cortex AT8-ir (Barnes et al., 2024; Datta et al., 2021; Paspalas et al., 2018), but no rhesus has yet been reported to harbor Braak stage V/VI tauopathy. Of these reports, Paspalas et al. (2018) identified AT8+ fibrils with an ultrastructure resembling human AD PHFs. Five reports have examined AT8-ir in cynomolgus macaques. Like rhesus, the hippocampus and temporal cortex were common sites of neuronal AT8-ir in cynomolgus aged ≥26 years (Oikawa et al., 2010; Uchihara et al., 2016). Additionally, one report described AT8-ir in the basal ganglia and neocortex of ≥30-year-olds. These same animals harbored AT8+ pretangles and oligodendrocyte-like cells that only colocalized with 4R-tau (Uchihara et al., 2016). Interestingly, diffuse AT8-ir was observed in neuronal soma of the hippocampus and prefrontal cortex of 14- to 19-year-olds cynomolgus (Jester et al., 2022). Among four reports, baboons aged ≥19 years were found to have neocortical AT8-ir in neurons and glia, preferentially in the medial temporal lobe, with substantial interindividual variation (Härtig et al., 2000; Ndung'u et al., 2012; Schultz et al., 2000a; Schultz et al., 2000b). Two of three reports in African green monkeys identified AT8-ir in animals aged >20 years (Cramer et al., 2018; Lemere et al., 2004). In two animals, AT8-ir occurred as pretangles in the entorhinal cortex and hippocampus (Cramer et al., 2018), and a third animal had sparse AT8-ir in neuritic plaques of the temporal cortex (Lemere et al., 2004). Atypically, one study found granular cytoplasmic AT8-ir in small cells throughout the neocortex, mainly the superior occipital gyrus, in the striatum, and rarely in the hippocampus of African green monkeys aged ≥8.2 years (Latimer et al., 2019). Lastly, one report of two guenons aged 27 and 30 years detected rare AT8-ir cells in the cingulate cortex (Härtig et al., 2000).

Other notable findings in Old World monkeys include PHF1-ir in neuritic amyloid plaques in rhesus aged ≥25 years (Shah et al., 2010), and the presence of AT8+, PHF1+, and TG3 + perivascular astroglial inclusions and oligodendroglial coiled bodies in the limbic system of a 30-year-old baboon. Approximately 10% of the tau inclusions in the baboon were argyrophilic (Schultz et al., 2000a). In a 35-year-old cynomolgus macaque, argyrophilic glial fibrillary tangles were found in the striatum, thalamus, midbrain, and brainstem (Kiatipattanasakul et al., 2000).

### 6.3 Aged New World monkeys

Ten studies have examined tau pathologies in New World monkeys, encompassing 65 common marmosets, 36 cotton top tamarins, and 38 squirrel monkeys between 1 and 24 years old (Table 5). Common marmosets presented AT100-ir and Alz50-ir in dystrophic microglia of 1- to 18-year-old common marmosets that increased with age (Rodriguez-Callejas et al., 2016). AT8-ir was reported as negative in one study of marmosets aged 7–10 years (Freire-Cobo et al., 2023). Silver staining has not been evaluated in marmosets. Cotton top tamarins and squirrel monkeys were negative for argyrophilic NFTs. Squirrel monkeys (number of subjects not disclosed) had occasional AT8+ and Alz50+ cortical neurons and neuritic plaques (Elfenbein et al., 2007).

### 6.4 Aged prosimians

In prosimians, six studies evaluated the brains of 163 gray mouse lemurs aged 1–13 years, and one report evaluated 2 Brown lemurs aged 12 and 14 years (Table 6). Of these studies, only one examined argyrophilic NFT occurrence in 25 gray mouse lemurs and reported negative results (Bons et al., 1992). AT8-ir has not been explored in gray mouse lemurs and was only reported in one 12-year-old Brown lemur (Härtig et al., 2000). Other reported tau-related findings include weak neocortical PHF tau in few gray mouse lemurs aged 7–13 years (Bons et al., 1995a; Bons et al., 1995b), and faint CP13-ir in a few scattered hippocampal cells of three gray mouse lemurs aged 6–11 years (Kraska et al., 2011).

### 6.5 Age- or disease-related tau pathologies in NHPs?

An analyses of the above listed reports supports the concept that aged chimps (Edler et al., 2017; Rosen et al., 2008; Rosen et al., 2011), rhesus (Paspalas et al., 2018), cynomolgus (Kiatipattanasakul et al., 2000; Oikawa et al., 2010; Uchihara et al., 2016), baboons (Schultz et al., 2000a; Schultz et al., 2000b), and African green monkeys (Cramer et al., 2018) can develop tau pathologies, identified as classic argyrophilic or AT8+ NFTs. Evidence of NFTs is still missing in aged gorillas, orangutans, Campbell’s guenon, common marmoset monkeys, squirrel monkeys, cotton top tamarins and lemurs (Tables 3–6). The number of subjects with tau inclusions is overall small but the overall number of aged subjects is also small.

A critical issue to consider is how an “aged animal” is defined, which depends on the NHP species. The interpretation of the results is affected by the chosen method to match ages between NHPs and humans, and whether the age matching considers lifespan and reproductive senescence (Figure 2). This issue is especially important for New World monkeys and prosimians, as it is currently unknown at what age females experience a decline in fertility and may have affected the detection of tau pathologies.

The clinical diagnosis of AD or FTD is complex and requires multimodal evaluations over time, including records of cognitive or executive and/or motor impairment, fluid biomarkers, and/or imaging. Misdiagnoses, especially between FTD and primary psychiatric disorders, are not uncommon (Ducharme et al., 2020). Diagnoses are ultimately confirmed postmortem by the presence of specific proteinopathies characterized by their neuroanatomical distribution, burden, and morphology. The sole postmortem identification of a few tau pathologies (or AT8-ir neurons) is not considered a diagnosis of tauopathy. Interestingly, most reports in old NHPs did not aim to identify tauopathies but rather to assess whether AD-like pathology was present as an indication of the animals developing or having AD.

Age-related neurological changes in NHPs such as cognitive and motor dysfunction (Emborg et al., 1998; Hopkins et al., 2021; Moore et al., 2006), brain atrophy on MRI (Kraska et al., 2011; Sridharan et al., 2012), variations in CSF and blood biomarkers (Darusman et al., 2014; Latimer et al., 2019; Metzler et al., 2025; Yue et al., 2014) and postmortem tau inclusions (listed above), have been documented. Yet, most studies have seldom evaluated multiple domains per animal in statistically powered projects due to the low availability of aged NHPs, high cost of multiple evaluations, and academic pressures to produce results. Opportunistic sampling of postmortem tissue has emerged as an efficient way to probe specific questions, albeit with the limitation of forfeiting a valid diagnosis of AD, FTD, or other neurodegenerative disease. For example, the identification of NFTs in >37-year-old chimps (Edler et al., 2017) demonstrate the vulnerability of the species to harbor tau pathology, although a tauopathy diagnoses cannot be asserted due to missing behavioral data.

Tauopathies may occur in NHPs. The 38-year-old macaque harboring NFTs identified by Paspalas et al. (2018) had “pronounced cognitive deficits” defined by the greater number of trials the animals needed to learn a delayed non-match-to-sample task compared to younger macaques. A caveat is that age-related decline is expected in NHPs, and this was the only advanced age animal tested in the study. Barnes et al. (2024) aimed to ascertain AD diagnoses in aged rhesus by applying the ABC and ATN diagnostic scales; the investigators combined postmortem results with *in vivo* cognitive and MRI data. Although the NHPs presented AT8-ir, none of the aged subjects had NFTs. The cynomolgus with PSP-like pathologies in the report by Kiatipattanasakul et al. (2000) had gait disturbance and involuntary trembling supporting a potential PSP disease condition that may differ from age-related decline. These findings suggest that NHPs could develop tauopathies; application of current standards of disease diagnoses is needed for confirmation. Overall, the question of whether NHPs can develop AD or FTD waits to be fully answered.

## 7 NHP models of tauopathy

Although some aged NHPs, like some aged humans, can develop disease-like pathology, the accumulated data show that investigators cannot rely on identifying enough affected monkeys to conduct studies powered for systematic evaluation of disease mechanisms or novel therapies. The alternative is to create NHP models of tauopathy by treating animals with different agents that may induce a disease-like condition.

To investigate currently available NHP models of tauopathies we expanded our PubMed search on aged NHP pathologies to include the keywords “models,” “Alzheimer’s disease” and “frontotemporal dementia.” We also examined reference lists in review articles to gather additional articles and focused on peer-reviewed articles in English. Fifteen articles that reported on the expression of tau were selected and analyzed to extract information on findings per species. See Tables 7–9 for compiled references and corresponding data on NHP models of tauopathy, with exception of three reports on transgenic models (discussed in section 7.4).

**Table 7:** Peer-reviewed publications intracerebral delivery of brain homogenates to NHPs.

Reference	Species	*n*	Treatment (brain homogenate source)	Injection site; dosing paradigm	Time to necropsy (yrs.)	Age at necropsy (yrs.)	AT8+, ThioS+, or Silver+ NFTs (n+/nTotal)	AT8-ir (n+/nTotal)	Other tau antibodies (n+/nTotal)	Behavior and notes
Baker et al. (1993)	common marmoset (*C. jacchus*)	3	1 early onset AD	L Cd and hpc, R NA and amg, BiL parietal ctx; 50 μL/site	6.4–8.7	8.3–8.5	0/3	NE	Tau (epitope NR): 0/3	Behavior: NE Notes: -
		8	3 suspected prion dementia		4.7–5.8	6–7.2	0/8	NE	Tau (epitope NR): 0/3	
		3	Non-injected	N/A	N/A	8.2–8.7	0/3	NE	Tau (epitope NR): 0/3	
Ridley et al. (2006)	common marmoset (*C. jacchus*)	9	3 sporadic AD	L Cd and hpc, R NA and amg, BiL parietal ctx; 50 μL/site	1.2–7.9	7.2–13.8	0/9	NE	Tau (epitope NR): 0/9	Behavior: NE Notes: -
		5	1 familial AD		0.9–6.4	5.9–16.5	0/5	NE	Tau (epitope NR): 0/5	
		6	2 Down Syndrome brains		3.9–8.1	6.3–11.0	0/6	NE	Tau (epitope NR): 0/6	
		2	1 Gerstmann-Straussler-Scheinker syndrome with Aβ		4.6	6–6.4	0/2	NE	Tau (epitope NR): 0/2	
		5	3 marmosets Aβ + previously treated with AD homogenate (Baker et al., 1993)		1.9–7.9	6.5–12.5	0/5	NE	Tau (epitope NR): 0/5	
		8	2 human CTL		0.8–9.5	7.1–13	0/8	NE	Tau (epitope NR): 0/8	
		3	2 carriers of different PrP mutations		5.7–5.8	7.2–7.3	0/3	NE	Tau (epitope NR): 0/3	
		10	CSF from non-demented patients with psychiatric disorders		2.5	~6.2	0/10	NE	Tau (epitope NR): 0/10	
		40	Non-injected	N/A	N/A	5–19	0/40	NE	Tau (epitope NR): 0/40	
Gary et al. (2019)	gray mouse lemur (*M. murinus*)	6*	2 AD	parietal ctx 4×10% w/v 13 μL	1.5	4.8–5.2	0/5	2/5; close to injection site, mainly NTs, also globular cells, horseshoe and punctiform accumulations, rare somatodendritic inclusions	MC1, AT100: 2/5; close to injection site, mainly NTs, also globular cells, horseshoe and punctiform accumulations, rare somatodendritic inclusions	Behavior: Visual discrimination task at 6 mpi, and accelerating rotarod at 0.5, 1, and 1.5 ypi, ND between groups; learning task at 1 and 1.5 ypi, AD-injected NHPs decline in performance Notes: *5 used for IHC. **Two CTL-injected NHPs euthanized at 1 ypi due to infection.
		6	Human CTL		1–1.5**	4.3–5.2	0/5	0/5	MC1, AT100: 0/5	
Darricau et al. (2022)	rhesus (*M. mulatta*)	2	Sarkosyl-insoluble tau from 2 PSP mesencephalons*	BiL supranigral area; 2x10μL 2 μg/mL	1.5	8.5	2/2; AT8+ supranigral area	2/2; tufted astrocytes, globose tangles, CBs, and NTs in supra-nigral area, thl (needle track), Cd, Pu, GP	4R-tau: 2/2 3R-tau: 0/2	Behavior: Kinematics analysis at 1 and 1.5 ypi, increase in step duration and decreases in step length, limb length, and articulation range of motion in PSP-tau NHPs; Object retrieval task: ND on “easy” trials, completion time, or # of motor errors, but more cognitive errors on “hard” trials in PSP-tau NHPs Notes: *PSP-tau from the mesencephalon was selected due to higher *in vitro* seeding activity vs. frontal ctx. **One CTL brain sample had Braak II tauopathy and Thal I amyloid
		2	Sarkosyl-insoluble tau from 2 CTL frontal ctx**	BiL supranigral area; 2x10μL < 200 ng/mL	1.5	8.5	0/2	0/2	4R-tau: 0/2 3R-tau: 0/2	
Darricau et al. (2024)	rhesus (*M. mulatta*)	4	Sarkosyl-insoluble tau from 4 AD + sham	AD-tau: BiL ec; 4x25μL 115 ng/mL Sham: BiL ventricles; 15% sucrose	1.5	14–17	0/4*	4/4; NTs in ec, hpc, cg	NE	Behavior: NE Notes: *Authors report no AT8+ NFTs in these cohorts, yet some presence is noted in the quantification graph
		3	Sarkosyl-insoluble tau from 4 AD + recombinant AβOs	AD-tau: BiL ec; 4x25μL 115 ng/mL/site AβOs: BiL ventricles; 100 μg total at baseline and 0.5 yr.	1.5	15–16	3/3; AT8+, ec, hpc, cg 2/3; AT8+, temporal ctx	1/3; tangles and NTs in ec, hpc, cg	NE	
		3	Sarkosyl-insoluble tau from 4 CTL + recombinant AβOs	CTL-tau: BiL ec; 4 4x200μL < 10 ng/mL, per hemisphere AβOs: BiL ventricles; 100 μg total at baseline and 0.5 yr.	1.5	14–16	0/3*	0/3	NE	
		4	Sarkosyl-insoluble tau from4 CTL + sham	CTL-tau: BiL ec; 4x200μL < 10 ng/mL, per hemisphere Sham: BiL ventricles	1.5	14–16	0/4*	0/4*	NE	
		3	Sham-operated	BiL ec and BiL ventricles; 15% sucrose	1.5	13–16	0/3	0/3	NE	

Results are reported as n positive NHPs to n total NHPs in the study (n+/nTotal); brain region and morphological description are included if available. *Details specific issues in the publication of the corresponding row denoted with corresponding asterisk(s). Aβ, amyloid beta; AβOs, amyloid beta oligomers; AD, Alzheimer’s disease; amg, amygdala; BiL, bilateral; CBs, coiled bodies; Cd, caudate; CSF, cerebrospinal fluid; CTL, control; ctx, cortex; GP, globus pallidus; hpc, hippocampus; L, left; NA, nucleus accumbens; N/A, not applicable; ND, no difference; NE, not evaluated; NT, neuropil thread; PrP, prion protein; PSP, progressive supranuclear palsy; Pu, putamen; R, right; ThioS+, Thioflavin S positive; thl, thalamus; w/v, weight to volume; ypi, years post-injection; yrs., years.

**Table 8:** Peer-reviewed publications reporting intracerebral delivery of synthetic Aβ peptides and oligomers (as described by authors) to NHPs.

References	Species	*n*	Treatment	Injection site; dosing paradigm	Time to necropsy (d or yrs.)	Age at necropsy (yrs.)	AT8+, ThioS+, or Silver+ NFTs (n+/nTotal)	AT8-ir (n+/nTotal)	Other tau antibodies (n+/nTotal)	Behavior and notes
Geula et al. (1998)	rhesus (*M. mulatta*)	1	Fibrillar Aβ40 peptide	Frontal, parietal, and anterior temporal ctx; 200 pg./1uL PBS	11-12 d	25–28	NE	NE	pS262-tau, PHF-1*: 1/1; neurons and neurites around injection site	Behavior: NE Notes: Native Aβ deposits and PHF-1-ir dystrophic neurites were present in aged rhesus ctx. *Results of p-tau IHC referred as p-tau-ir **Aβ species NR ***Inconsistencies between text and figures on n animals per treatment
		1	Fibrillar Aβ42 peptide		11-12 d	25–28	NE	NE	pS262-tau, PHF-1*: 1/1; results not shown, neurons and neurites around injection site	
		1	Soluble Aβ** peptide		11-12 d	25–28	NE	NE	pS262-tau, PHF-1*: 0/1; insignificant	
		1	Vehicle		11-12 d	25–28	NE	NE	pS262-tau, PHF-1*: 0/1; insignificant	
		1	Fibrillar Aβ** peptide		11-12 d	5	NE	NE	pS262-tau, PHF-1*: 0/1; insignificant	
		1	Soluble Aβ** peptide		11-12 d	5	NE	NE	pS262-tau, PHF-1*: 0/1; insignificant	
	common marmoset (*C. jacchus*)	5	Fibrillar Aβ** peptide		11-12 d	8–10	NE	NE	pS262-tau, PHF-1*: NR/5; significant	
		5	Fibrillar Aβ** peptide		11-12 d	2–3	NE	NE	pS262-tau, PHF-1*: 0/5; insignificant	
	?***	?***	Fibrillar Aβ** peptide	Frontal, parietal, and anterior temporal ctx; 20 ng/1uL PBS	11-12 d	2–3	NE	NE	pS262-tau, PHF-1: neurons and neurites distal to area of neuronal loss	
Ridley et al. (2006)	Common marmoset (*C. jacchus*)	2	Synthetic Aβ1-40 peptide	L Cd and hpc, R NA and amg, BiL parietal ctx; 50 μL/site	9.9-10.1 yrs.	13.1–14.7	0/2	NE	Tau (epitope NR): 0/2	Behavior: NE Notes: -
		2	Synthetic Aβ1-42 peptide		3.3–6.1 yrs.	7.8–10.1	0/2	NE	Tau (epitope NR): 0/2	
		2	Synthetic Aβ40-1 peptide		4.8–8.8 yrs.	7.4–10.5	0/2	NE	Tau (epitope NR): 0/5	
Forny-Germano et al. (2014)	Cynomolgus (*M. fascicularis*)	4	AβOs from Aβ1-42 peptide	lateral ventricles; 10-100 μg AβOs every 3 d for up to 24 d	7 d	9 or 16	0/4	rep NHP + in frontal ctx	pS396-tau, AT100, CP13; 4/4, frontal ctx, hpc, amg MC1: rep NHP + in neuronal soma of frontal ctx PHF1, Alz50: rep NHP + in frontal ctx	Behavior: NE Notes: CP13, AT100, and pSer296-tau all increased in AβO-injected NHPs compared to sham-operated.
		3	Sham-operated non-injected	N/A	7 d	9 or 16	NR	NR	pS396-tau: 3/3, frontal ctx, hpc DG, amg MC1, AT100, PHF1, Alz50: 0/3 CP13: 3/3, frontal ctx, hpc, amg	
Yue et al. (2021)	cynomolgus (*M. fascicularis*)	7	Synthetic AβOs from Aβ1-42 peptide	BiL WM adjacent to the dorsal and lateral hpc; total 800 μg AβOs (0.5 μg/μL) at baseline, 45 d, 90 d, and 150 d	240 d-1 yr.	20–22	6/7; AT8+, pfc, ec, parietal ctx, temporal ctx, hpc, striatum, silver+ in parietal and temporal ctx	6/7*; neurons and glia in pfc, ec, astrocytes in medium septum, both neurons and astrocytes in parietal ctx, temporal ctx, hpc, striatum	AT100: rep images of tangles and neurons in temporal and frontal ctx, hpc, thl, glia in parietal ctx	Behavior: NE Notes: *AT8+ tangles reported in 6/7 NHPs, unclear if 7^th^ NHP had AT8-ir without tangles **AT8+ tangles reported in 2/3 NHPs; unclear if 3^rd^ NHP had AT8-ir without tangles
		3	Synthetic AβOs from Aβ1-42 peptide	BiL WM adjacent to the dorsal and lateral hpc; 200 mg/side for 15 d	150 or 270 d	20–24	2/3; AT8+ temporal ctx	2/3**; temporal and parietal ctx	NE	
		3	Synthetic AβOs from Aβ1-42 peptide	BiL lateral ventricles; 200 mg/side, for 10 d	150 or 270 d	20–22	0/3	0/3	NE	
		7	Non-injected	N/A	N/A	18–20	0/7	0/7	AT100: listed, results NR	
Wakeman et al. (2022)	African green monkey (*C. aethiops sabaeus*)	2	AβO	R lateral ventricle; 100 or 200 μg delivered 3x7 d for 28 d	7 d	9.3–10.5	0/2	2/2*; hpc	Alz50, CP13: listed, results NR	Behavior: NE Notes: *AT8-ir reported to be increased compared to vehicle-injected NHPs in the MTL. **Methods lists 23 injected NHPs, results show data from 22 NHPs. AT8-ir reported to be increased in ec compared to vehicle-injected NHPs ***Image shows limited AT8-ir in a rep vehicle-injected subject. ****Methods lists 7 vehicle-injected NHPs, results show data from 8 NHPs *****CTLs for ICV NHPs ******CTLs for IT NHPs
		23**	AβO	IT at thoracolumbar junction; 100 or 200 μg either 1x7 d or 3x7 d for 28 d	7, 28, or 84 d	9.3–10.5	0/22*	22/22**; hpc, ec	Alz50: 0/22 CP13: rep NHP + in hpc and ec	
		2	Vehicle (2% DMSO in PBS)	R lateral ventricle; 100 or 200 μg 3x7 d for 28 d	7 d	9.2–10.4	0/2	2/2***; hpc	Alz50, CP13: listed, results NR	
		7****	Vehicle (2% DMSO in PBS)	IT at thoracolumbar junction; 100 or 200 μg either 1x7 d or 3x7 d for 28 d	7, 28, or 84 d	9.2–10.4	0/8*	8/8****; hpc, ec	Alz50: 0/8 CP13: rep NHP + in hpc	
		5*****	Non-injected	N/A	N/A	9.2–10.4	NR	NR	Alz50, CP13: listed, results NR	
		2******	Non-injected	N/A	N/A	9.2–10.4	NR	NR	Alz50, CP13: listed, results NR	

Results are reported as number of positive NHPs to total number of NHPs in the study (n+/nTotal); brain region and morphological description are included if available. *Details specific issues in the publication of the corresponding row denoted with corresponding asterisk(s). Aβ, amyloid beta; AβOs, amyloid beta oligomers; amg, amygdala; BiL, bilateral; Cd, caudate; CTL, control; ctx, cortex; d, days; DMSO, dimethyl sulfoxide; hpc, hippocampus; −ir, immunoreactivity; L, left; MTL, medial temporal lobe; NA, nucleus accumbens; N/A, not applicable; NE, not evaluated; NR, not reported; NT, neuropil thread; PBS, phosphate buffered saline; R, right; rep, representative; ThioS+, Thioflavin S positive; thl, thalamus; yrs., years.

**Table 9:** Peer-reviewed publications reporting intracerebral delivery of viral vector to NHPs.

References	Species	*n*	Treatment	Injection site; dosing paradigm	Time to necropsy (d)	Age at necropsy (yrs.)	AT8+, ThioS+, or Silver+ NFTs (n+/nTotal)	AT8-ir (n+/nTotal)	Other tau antibodies (n+/nTotal)	Behavior and notes
Beckman et al. (2021)	Rhesus (*M. mulatta*)	4	AAV1-P301L/S320F; AAV1-GFP	AAV1-P301L/S320F: L ec; 1.176×10^13^ genomic copies/mL AAV1-GFP: R ec; 1.6×10^13^ genomic copies/mL	90 d	10–15	4/4; hpc, retrosplenial ctx, L ec	4/4; hpc, BiL ec, retrosplenial ctx, V4 and V1 in occipital lobe	AT100, pS422-tau: rep NHP + in L ec pS262-tau, pThr231-tau, TOC1: rep NHP + in BiL hpc pS214-tau: rep NHP + in L hpc PHF1, MC1: rep NHP + in hpc	Behavior: NE Notes: -
		4	Non-injected	N/A	N/A	10–14	NR	NR	TOC1, Alz50, pS422-tau, TNT2, Tau5, TauC3, PHF1, MN423: 0/4	
		4	Aged non-injected	N/A	N/A	21–25	NR	NR	TOC1, Alz50, pS422-tau, TNT2, Tau5, TauC3, PHF1, MN423: 0/4	
Beckman et al. (2024)	Rhesus (*M. mulatta*)	8	AAV1-P301L/S320F	L ec; 2x18 μL, 1.176×10^13^ genomic copies/mL	90 or 180 d	10–16	8/8; AT8+ and ThioS+ in ec, hpc retrosplenial ctx, inferior temporal gyrus	8/8; pretangles in ec, hpc, retrosplenial ctx, inferior temporal gyrus	TOC1: rep. NHP + in temporal lobe Alz50, pS422-tau: rep NHP + in hpc/ec TNT2, Tau5, TauC3, PHF1: rep NHP +, region NR MN423: 180 d rep NHP +	Behavior: NE Notes: -
		4	AAV1-CTL	L ec; 2x18 μL viral load NR	90 or 180 d	10–16	0/4	0/4	TOC1, Alz50, pS422-tau, TNT2, Tau5, TauC3, PHF1, MN423: 0/4	
Jiang et al. (2024)	Rhesus (*M. mulatta*)	7	rAAV9 CAG::FRT-hTau + rAAV9 Syn::FLPo + AAV9 Syn::GFP	BiL hpc; 6×7.5 μL, 10^11–12^ genomic copies/mL per hpc	42–70 or 350 d	7–16	0/7*	rep image of AT8+ at 42 dpi in hpc; n/7 AT8+ NHPs NR	pT231-tau: rep image at 6 wpi in hpc	Behavior: Spatial working memory (*n* = 5) at 56 dpi, significant impairment in hTau NHPs; Delayed match to sample test (*n* = 5) at 56 dpi, significant impairment in hTau NHPs Notes: *The authors describe AT8+ pre- and mature NFTs, yet images only show AT8+ neurites and soma
		4	rAAV9 CAG::FRT-hTau + rAAV9 Syn::FLPo		42–70 or 350 d	10–15	0/4	rep image of AT8+ at 42 dpi in hpc	pT231-tau: rep image at 6 wpi in hpc	
		2	rAAV9 CAG::FRT-hTau + AAV9 Syn::FLPo		42–70 or 350 d	7–8	0/2	0/2	pT231-tau: 0/2	
		5	rAAV9 CAG::FRT-hTau		42–70 or 350 d	7–15	0/5	0/5	pT231-tau: 0/5	

Results are reported as n positive NHPs to n total NHPs in the study (n+/nTotal); brain region and morphological description are included if available. *Details specific issues in the publication of the corresponding row denoted with corresponding asterisk(s). AAV, adeno-associated virus; BiL, bilateral; CTL, control; ctx, cortex; d, days; dpi, days post-injection; ec, entorhinal cortex; GFP, green fluorescent protein; hpc, hippocampus; −ir, immunoreactivity; L, left; N/A, not applicable; NE, not evaluated; NR, not reported; R, right; rep, representative; ThioS+, Thioflavin S positive; yrs., years.

Most publications focused on tauopathies secondary to A*β* pathology. Only three peer-reviewed reports (Beckman et al., 2021; Beckman et al., 2024; Darricau et al., 2022) utilized methods to produce primary tauopathies in NHPs. Methods for generating NHP models included intracerebral delivery of brain homogenates or protein extracts from patients, synthetic Aβ oligomers (AβOs), or viral vectors to increase Aβ or tau burden in adult animals. A similar concept of modeling by increasing Aβ burden has been applied by combining viral vectors and reproductive technologies to generate transgenic NHPs carrying disease-related mutations. All studies performed postmortem pathological evaluation; antemortem behavioral studies were rarely conducted. As described in the section for aged NHPs, our criteria for NFT detection in a publication on NHP models of tauopathy was the authors’ description of the intraneuronal inclusion positive for silver and/or AT8 staining, with a corresponding image.

### 7.1 Models based on intracerebral delivery of brain homogenates or protein extracts

Starting in the early 1990s, five studies explored whether tauopathies developed after intracerebral delivery of brain homogenates from patients with a range of conditions to common marmosets, lemurs, and rhesus (Baker et al., 1993; Darricau et al., 2024; Darricau et al., 2022; Gary et al., 2019; Ridley et al., 2006) (Table 7).

Baker et al. (1993) produced the first publication aiming to induce AD pathologies in marmosets. The investigators injected three 2–3 years old marmosets in the left caudate and hippocampus, the right nucleus accumbens and amygdala, and both parietal cortices with brain homogenates from a single patient with early onset AD. At necropsy, approximately 6.5 years later, the marmosets presented Aβ plaque-like pathologies and occasional angiopathy in the cerebral cortex and amygdala, but not in deeper brain structures. NFTs were not detected; the antibody utilized for tau identification was not specified.

A follow-up study by Ridley et al. (2006) described injecting marmosets (<10 years old or >10 years old) in the same intracerebral sites as the Baker study with various brain homogenates. Nine marmosets were injected with samples obtained from three sporadic AD cases, five marmosets were injected with homogenate from one familial AD case attributed to a codon 717 mutation in APP, six marmosets were injected with samples obtained from two cases of cerebral amyloid angiopathy (CAA) associated with Down syndrome, and two marmosets were injected with homogenate from one case of spongiform encephalopathy associated to a prion protein gene mutation; this brain also contained cerebral β-amyloidosis (Ridley et al., 2006). The investigators also injected five animals with brain homogenates from three marmosets previously injected with an AD brain homogenate; these subjects were part of the above-mentioned Baker et al. (1993) study. Of the 27 injected marmosets, 24 displayed differing degrees of Aβ plaques and CAA in the brain at >6 years post-injection. The three monkeys without Aβ pathology had received brain homogenates from a sporadic AD case, a familial AD case, and a previously injected marmoset, respectively. None displayed NFTs or tau-ir; as in the previous publication, the antibody utilized for tau detection was not specified.

More recently, Gary et al. (2019) reported the effects of injecting six young (3.5-year-old) grey mouse lemurs in four sites surrounding the parietal cortex with brain homogenates from AD patients; an additional six lemurs of the same age were injected with brain homogenates from healthy patients. One year and a half later, the AD brain-inoculated lemurs displayed progressive cognitive impairment but no motor dysfunction, abnormal electroencephalograph signals, and cerebral atrophy on MRI. Postmortem, Aβ plaques or CAA were detected in all five AD-inoculated animals; phosphorylated tau evaluated by AT8, MC1, and AT100 immunostaining, was only detected in two of these animals, NFTs were not found. Interestingly, the authors noted that the two animals with phosphorylated tau immunoreactivities displayed the greatest hippocampal neuronal loss and worst memory scores. Also of interest, the animal that developed CAA was inoculated with the only AD brain homogenate that displayed CAA, suggesting that different protein strains may induce differing pathology.

Using a different brain homogenate treatment, Darricau et al. (2022, 2024) produced two consecutive reports on rhesus macaques. In the first study (Darricau et al., 2024; Darricau et al., 2022), two ~7 years old rhesus monkeys were injected into the supranigral area with sarkosyl-insoluble tau seeds extracted from the frontal cortex and mesencephalon of two PSP patients, one with pure PSP pathology, and one with a combination of PSP and Braak stage II AD tauopathy. Two additional rhesus macaques received homogenates from the frontal cortex of two control brains, one of which was devoid of pathology and the other with Braak stage II tauopathy and Thal stage 1 amyloid deposition. It should be noted that each monkey was injected solely with one single donor brain extract (PSP or control). At 6 months post-injection, the two macaques that received PSP seeds displayed parkinsonian-like motor impairments including increased step duration, decreased step length, and decreased range of motion. They also performed worse than the control macaques on “harder” trials of an object retrieval task. The animals were euthanized at 1.5-years post-injection and AT8-ir was observed in the supranigral area, ventral thalamus, substantia nigra, and globus pallidus of the PSP-injected macaques. Importantly, tau pathologies presented as NFTs, neuropil threads, tufted astrocytes, and coiled bodies; only 4R tauopathies were observed. In the second publication (Darricau et al., 2024; Darricau et al., 2022), macaques received purified tau extracted from brains of AD patients (AD-tau) with or without synthetic amyloid-β oligomers (AβOs). AD-tau injections induced AT8+ neuropil threads in the hippocampus, entorhinal cortex, and cingulate cortex at 1.5 years post injection. NFTs were only observed in macaques that received AD-tau plus AβOs.

The studies utilizing intracerebral injection of brain homogenates from patients demonstrate the feasibility of testing the pathogenic potential of a specific sample and its capacity to spread the affected proteins in a prion-like manner to interconnected brain regions. Issues associated to neurosurgical technique, e.g., brain targeting accuracy and backflow of infusate through the needle track, may affect protein distribution and accumulation and needs to be considered for interpretation of the results. For modeling purposes, the variability across donor tissues and sample preparation can influence the replicability of the syndrome; biochemical characterization of donor tissue prior to injection may help overcome this issue. Of the 5 studies utilizing human brain homogenates for disease modeling in NHPs (Table 7), only the two reports by Darricau et al. removed potentially seed-competent contaminants from their donor sources by performing sarkosyl-insoluble tau extractions from PSP and AD patients, respectively (Darricau et al., 2024; Darricau et al., 2022).

### 7.2 Models based on CNS delivery of aβ peptides and synthetic AβOs

In addition of the above-mentioned report by Darricau et al. (2024) that combined AD-tau with AβOs, five studies tested the effects of Aβ peptides or AβOs (as reported by the authors) in marmosets, rhesus macaques, cynomolgus macaques, and African green monkeys (Forny-Germano et al., 2014; Geula et al., 1998; Ridley et al., 2006; Wakeman et al., 2022; Yue et al., 2021) (Table 8).

In the first of these reports by Geula et al. (1998), the investigators intracerebrally injected fibrillar Aβ to one 5- and two 25-28-year-old rhesus macaques. After 11–12 days, diffuse Aβ accumulation sometimes accompanied by a core structure was present at the injection site of the older macaques, as well as an area of neuron loss extending up to 1.5 mm from the injection site. pSer262-tau-ir neurons and neurites were also present up to 2 mm from the injection site. Comparatively, the 5-year-old macaque had limited p-tau-ir. Injections of soluble Aβ40 and PBS vehicle also did not produce a robust response. The investigators also injected fibrillar Aβ to young (2-3 yrs) and aged (8-10 yrs) common marmosets. The aged marmosets had Aβ accumulation and p-tau-ir, although less than the aged macaques. Silver staining and AT8-ir were not evaluated.

The above-mentioned report by Ridley et al. (2006) included a group of six marmosets that were injected with AβOs in the left caudate and hippocampus, the right nucleus accumbens and amygdala, and both parietal cortices. No NFTs or Aβ plaques were found in the monkeys for up to 10 years post-injection.

Later, Forny-Germano et al. (2014) delivered AβOs to the lateral ventricles of 9- and 16-year-old cynomolgus macaques. Injections were given once every 3 days for up to 24 days. Seven days after the final injection, AβOs were detected in neurons of the cortex, hippocampus, striatum, and amygdala. The authors also reported an increase in pSer396-tau, AT100, and CP13 immunopositivity in these regions, as well as increased AT8, Alz50, and PHF-1 immunopositivity in the frontal cortex. Interestingly, thioflavin-S-positive NFTs were observed in the neocortex. Immunogold electron microscopy with antibodies MC-1, Alz50, and PHF-1 confirmed the presence of tau filaments in the frontal cortex. NFTs were not detected.

Yue et al. (2021) also treated cynomolgus macaques with AβOs. Seven 20–22-year-old monkeys received bilateral AβO injections above the hippocampus between the lateral basal ganglia and medial temporal lobe at four different time points over 150 days. At >240 days after the last injection, all seven monkeys developed Aβ plaques in a similar spatial distribution, though plaque density varied greatly between subjects. Notably, two of the AβO-injected macaques had a similar number of plaques in the brain as non-injected control subjects. All but one AβO-injected monkey developed NFTs and NTs in several brain regions that were labeled by silver, AT8, and AT100.

Lastly, Wakeman et al. (2022) injected 9-10-year-old male African green monkeys with AβOs either via the intrathecal (*n* = 23) or intracerebroventricular (*n* = 2) route. Intrathecal dosing was given either one or three times per seven-day period for 28 days total, and the animals were euthanized either 7, 28 or 48 days after the last injection. Intracerebroventricular dosing was done three times per seven-day period for 28 days, and the animals were euthanized 7 days after the last injection. The investigators reported increased AT8-ir in the medial temporal lobe for both delivery methods, but the only statistically significant finding was increased AT8+ cells in the outer layers of the entorhinal cortex following intrathecal injections. Overt Aβ plaques or NFTs were not observed.

NHP models based on the injections of synthetic protein seeds present an opportunity to study prion-like spread of pathologies between interconnected brain regions. Similar to injections of brain homogenate neurosurgery-related issues need to be considered for interpretation of protein distribution. As investigators control the characteristics of the injected material, this method has the potential to generate replicable results and can be advantageous for modeling and assessing pathogenicity of different protein strains. The five available reports on this model injected various Aβ strains to NHPs with variable tau-related outcomes. NFTs were only detected in cynomolgus macaques that received 800 μg AβOs and were evaluated 240 days to 1 year later (Yue et al., 2021), but not with shorter period of times, e.g.: 7 days (Forny-Germano et al., 2014) or 84 days (Wakeman et al., 2022). These results suggest that an extended period of time may be required for NFT generation after AβOs. Thus far, no group has reported injection of tau seeds into NHPs.

### 7.3 Models based on intracerebral delivery of viral vectors

Three reports utilized intracerebral delivery of viral vectors to overexpress mutated or wild type tau protein in rhesus macaques (Beckman et al., 2021; Beckman et al., 2024; Jiang et al., 2024) (Table 9).

Beckman et al. (2021) injected four rhesus aged 10–15 years in the left entorhinal cortex with an adeno-associated virus serotype 1 vector encoding for 0N4R tau with the P301L/S320F mutations (AAV1-P301L/S320F). The same animals also received an AAV-green fluorescent protein (AAV1-GFP) injection in the contralateral entorhinal cortex; eight additional non-injected animals were used as young and aged controls. The authors proposed this approach for modeling AD, although tau mutations P301L and S320F are each independently linked to genetic FTD (Strang et al., 2019). Ninety days post-AAV delivery, the animals were euthanized. CSF and plasma collected antemortem showed significant increases in neurofilament light chain (NfL), total tau, sTREM2, TNFα, IL-6, pS199-tau, and pS396-tau. In the entorhinal cortex and hippocampus ipsilateral to AAV1-P301L/S320F delivery, many neurons were AT8+. Furthermore, NFTs (ThioS+/NeuN+) and ghost tangles (ThioS+/NeuN-) were detected in the hippocampus. In the contralateral, AAV-GFP-treated hippocampus, fibrillary tau pathology was not detected, although pSer262-tau-ir and pThr231-tau-ir were observed. Immunofluorescence revealed colocalization of 3R-tau-ir and 4R-tau-ir with AT8-ir in both hippocampi and entorhinal cortices. The authors proposed that the colocalization of AT8-ir and 3R-tau-ir was indicative of permissive templating of endogenous 3R-tau by the mutated exogenous 4R-tau and prion-like spread to interconnected brain regions. AT8-ir was also detected in the occipital lobe, retrosplenial cortex, and visual regions V4 and V1, suggesting protein spread from the injection site to interconnected brain regions. It should be noted that images from control brains were not provided.

A follow up study by Beckman et al. (2024) injected eight rhesus aged 10–16 years in the left entorhinal cortex with the same double-mutant 0N4R tau construct (AAV1-P301L/S320F). Four additional monkeys received an empty vector control (AAV1-CTR) in the left entorhinal cortex. Half of the animals were followed for 90 days, and the other half for 180 days. Antemortem CSF biomarkers were elevated in the AAV1-P301L/S320F group beginning at different time points post-injection; pS199-tau at 30 days, total tau and pS396-tau at 60 days, pT231-tau at 90 days, and pT181-tau at 120 days. NfL was increased in CSF at 30 days and serum at 60 days, while Brain Derived Neurotrophic Factor was decreased in CSF at 60 days and in serum at 90 days. In AAV1-P301L/S320F animals, antemortem imaging at 90 and 180 days detected a decreased in left hippocampal volume by MRI and a significant increase in uptake of tau radiotracer [18F]APN-1607 by PET. Postmortem, these monkeys had AT8+ pretangles in the left hippocampus and entorhinal cortex, as well as in the right entorhinal cortex that were more frequent in the 180 days group. They also had mature NFTs (AT8+/ThioS+/NeuN+) and ghost tangles (AT8-/ThioS+/NeuN-) in these regions that were also more frequent in the 180 days group. Similar to the investigators’ previous report, colocalization of AT8-ir and 3R-tau-ir was observed in the left entorhinal cortex and hippocampus, especially in the 180 days group. Several other tau immunoreactivities, including TOC1, Alz50, pS422-tau, TNT2, Tau5, TauC3, PHF1, MN423 were also detected.

Lastly, Jiang et al. (2024) overexpressed human 4R-tau in the hippocampi of 11 adult rhesus macaques (aged 7–16 years) using FLP/FRT-mediated recombination. The animals were injected with an AAV9 vector expressing a flippase recognition site (FRT) and 4R-tau under control of a CAG promotor (rAAV9 CAG::FRT-hTau), as well as an AAV9 vector expressing the recombinase flippase (FLPo) under a human synapsin promoter (rAAV9 Syn::FLPo). Seven additional animals received control injections of AAV9 CAG::FRT-hTau, which cannot express human tau without FLPo/FRT-mediated recombination. A subset of the tau-expressing (*n* = 7) and controls (*n* = 2) animals also received AAV9 Syn::GFP in the hippocampus. At 42 days post-injection, the tau-expressing animals displayed hippocampal atrophy on MRI, decreased metabolic activity by [18F]Fluoro-deoxy-glucose-PET, and increased uptake of the tau radiotracer [18F]T807 on PET. At 56 days, these monkeys showed impairments on spatial working memory and delayed match-to-sample tests compared to their preoperative baseline performance and control animals. Additionally, at 84 days they had increased CSF levels of total tau, pT181-tau, pT231-tau, NfL, and decreased Aβ_42_ compared to controls. The monkeys were euthanized at either 42-70-days or 350-days post-injection; the number of animals per time point is unclear. AT8-ir and pT231-tau-ir were detected in hippocampal neurites and soma in a 42-day tau-overexpressing monkey. Further histological analyses identified increased 3R- and 4R-tau-ir and neuron loss (indicated by reduced NeuN-ir and Nissl) in the hippocampus of tau-overexpressing subjects compared to controls.

Models generated by viral vector gene transfer increase protein burden by overexpression of the encoded protein. Reliable viral vector production and quality control of the produced titer is critical for replicability of the approach. Studies on protein transmission relying on these types of models should consider differences in anterograde or retrograde transport capabilities of viral vectors subtypes that could contribute to protein spreading and therefore confound the results, in addition to intracerebral targeting accuracy and/or backflow of infusate through the needle track. Viral vector gene transfer methods are particularly advantageous for studying disease-related mutations and their associated changes in the transcriptome and interactome. Careful selection of the gene (or part of the gene) to be overexpressed is critical for model development. Beckman et al. (2021, 2024) created an NHP model expressing double tau mutations P301L and S320F and Jiang et al. (2024) overexpressed non-mutated human 4R-tau. NFTs were only identified by Beckman and colleagues mainly at 180 days, underscoring the importance of time for the development of tau aggregates.

### 7.4 Transgenic models

Three reports utilized gene transfer of mutant human *amyloid precursor protein* (*APP*) gene to gametes or embryos to create transgenic models of AD in cynomolgus, rhesus, and common marmoset monkeys (Chan et al., 2022; Seita et al., 2020; Yoshimatsu et al., 2022).

Seita et al. (2020) transfected oocytes of cynomolgus monkeys with lentiviral vectors encoding for *APP* with either the Swedish mutation K595N/M596L, the Arctic mutation E618G, or the Iberian mutation I641F. Two female transgenic macaques were birthed; their plasma levels of Aβ_40_ and Aβ_42_ were 2- and 50-fold higher, respectively, compared to a wild type monkey. Two transgenic fetuses were aborted at embryonic days 101 and 102; the aborted fetuses had increased insoluble Aβ_42_ compared to an aborted wild type fetus. Neither Aβ-ir nor p-tau AT8-ir accumulation was detected in the fetal brain tissue.

Chan et al. (2022) also utilized lentiviral vectors to generate AD-like transgenic monkeys. The investigators transfected rhesus macaque oocytes with a lentiviral vector encoding for human *APP* carrying Swedish mutation K670M/N671L and Indiana mutation V717F. Two female transgenic rhesus were birthed and followed for 10 years. No volumetric brain differences were detected by MRI between *APP* mutation carriers and wild type controls. CSF levels of Aβ_42_, total tau, and pT181-tau were measured at 2, 4, 6, 8, and 9 years of age. Transgenic monkeys had elevated Aβ_42_ at 4 years of age and increased total tau at 2 and 4 years of age, but no difference in pT181-tau levels. Behaviorally, the transgenic animals presented some anxious behaviors and executive function deficiencies. At necropsy, only one of the transgenic monkeys displayed Aβ plaque deposition and CAA in the neocortex. CP13-ir was not detected.

Yoshimatsu et al. (2022) generated two transgenic marmosets harboring human *APP* with Swedish mutation K670M/N671L and Indiana mutation V717F via lentiviral transduction to pronuclear stage embryos. Genomic analysis showed that these transgenic marmosets were identical twins, yet one of the marmosets harbored ~3-fold more transgenic cells than the other. Postmortem analysis was performed at 7 years of age. One of the two marmosets had small Aβ plaque-like structures in the prefrontal cortex. Neither marmoset had AT8-ir.

Transgenic animals overexpressing pathogenic mutations present an opportunity for comprehensive disease modeling and for assessing pathogenesis across the lifespan of the monkeys; cohorts of animals could be generated by reproduction of mutated carriers. This approach presents many challenges associated to the availability of animals devoted to *in vitro* fertilization, and the complexity of the methods (for review see Schmidt et al., 2022). Furthermore, the development of the disease may require years, hampering research progress. Seita et al. (2020), Chan et al. (2022), and Yoshimatsu et al. (2022) generated transgenic macaques carrying multiple *APP* mutation; tauopathies were not identified probably because the monkeys were young at the time of necropsy. The generation of models with multiple mutations may speed up the development of pathology, yet it is not the most clinically relevant as multiple mutations are rare in human populations. Thus far, no groups have reported germline or embryonic modification of *MAPT* in NHPs.

## 8 Final thoughts

As stated in the introduction, the main aim of this article was to analyze available reports on tau pathologies and models of tauopathies in NHPs, considering the complexity of the tau protein and associated tau pathologies. Our exploration identified gaps in knowledge, as well as differences in the methods of evaluation and the criteria applied to identify pathologies and define a pathological condition across human and NHP species. In this final section, we discuss how to maximize current NHP tauopathy models, emerging paradigms for generating new models, and the ethical challenges associated with the study of tauopathies in NHPs.

### 8.1 Increasing the translational value and validity of current NHP models of tauopathy

NHP models of tauopathies are urgently needed for studying disease mechanisms and test first-in-class and invasive therapies. Recognizing the advantages and limitations of the different models is the first step toward identifying the best match to answer a scientific question.

Old NHPs are ideal models for the study of aging and tau pathologies (section 6) and may be more susceptible to experimentally induced tauopathies. Some animals may develop disease-like tau pathology; few reports have also documented behavioral changes that combined with the pathology may indicate primary or secondary tauopathy (Kiatipattanasakul et al., 2000), a possibility that requires systematic investigation. Addressing the gaps in knowledge regarding both human (section 2) and NHP (section 3) tau in physiological conditions would further shed light on the susceptibility of NHPs to tau-related disorders (Walker and Jucker, 2017). It is notable that marmosets and lemurs, unlike Old World monkeys, did not present tau inclusions with age, intracerebral injections of AD brain homogenates, or fibrillary Aβ. This finding underscores that differences in tau sequence across NHP species, albeit small, may affect pathophysiology and need to be considered for model development.

Accumulated data show that investigators cannot rely on identifying enough affected old NHPs to conduct statistically powered studies. As an alternative, intracerebral injection of either brain homogenates, protein extracts, or viral vectors have been utilized to locally increase the protein burden as a shortcut to induce pathology; attempts to transgenic modeling also applied the concept of increase protein burden. Most reports aimed to develop AD models by increasing Aβ burden. The exceptions utilized brain tissue from PSP patients (Darricau et al., 2024; Darricau et al., 2022) or viral vectors encoding for multiple tau mutations (Beckman et al., 2021; Beckman et al., 2024; Jiang et al., 2024). As understanding of tauopathies deepens, the models as well as the methods of evaluation need to be refined. In that regard, new models focused on increasing specific strains of tau burden resembling different primary tauopathies would be beneficial to the field.

Based on our review of the literature, we identified a number of parameters that could help evaluate NHP models of tauopathies as well as answer whether some NHPs can spontaneously develop tauopathies, especially AD. First, if identification of AT8+ NFTs is the standard for Braak staging in human disease (Braak et al., 2006), identification of tauopathy in NHPs should include AT8 immunohistochemistry. Second, when reporting AT8-ir pathology, a systematic evaluation of multiple regions in all study subjects, specifically in the transentorhinal, limbic, and isocortical regions is recommended; ideally a table should be presented to depict pathological burden per region for each animal. Quantification of filamentous tau inclusions and knowledge of their ultrastructure (e.g., PHFs) would help provide clarity in NHP studies. Third, reports of AD-like tau pathology would benefit from applying similar rigor of evaluation for detecting Aβ pathology (e.g., Thal scoring). Fourth, evaluation of neurodegeneration patterns, cell type affected and expression of 3R- and 4R-tau, alpha-synuclein and TDP-43, between others, would facilitate differential diagnoses. Fifth, *in vivo* behavioral, imaging, and biomarker data are needed to inform the postmortem analyses with clinically relevant measures to ultimately diagnose NHPs with tau-related diseases.

### 8.2 Emerging paradigms

Advances in biotechnology present opportunities for creating a new generation of NHP models of tauopathies. For example, viral vectors that can cross the blood brain barrier, such as AAV-CAP-B10 (Goertsen et al., 2022), could be used for introducing mutated *MAPT* genes without the need for intracerebral injections.

As an alternative to transgenesis, genome editing is envisioned to generate animal models that genocopy and phenocopy genetic disease conditions (Schmidt et al., 2022). Rizzo et al. (2023) have reported the successful development of marmosets carrying *PSEN1* point mutations C410Y and A426P by CRISPR/Cas9. The group predicts that the mutant marmosets will display Aβ accumulation detectable by PET by 4 years of age, equivalent to a 32-year-old human. The future results of this study may provide clues on whether marmosets develop true neurodegeneration with Aβ and tauopathy.

Targeted genotyping of NHPs with abnormal phenotypes or systematic genotyping of NHP colonies could help identify animals carrying mutations of interest. For example, a collaborative effort from several primate research centers reported whole genome sequencing of 84 common marmosets that identified 27 total variants with a clinical significance of pathogenic and/or likely pathogenic (Harris et al., 2023). Some of these variants occurred in AD risk genes, including a stop-gain variant in *ABCA7*. Whole genome sequencing of NHP colonies is pragmatic; it would inform whether coding and non-coding single nucleotide polymorphisms are conserved between humans and NHPs. Whole genome datasets are available from multiple NHP species (Kuderna et al., 2023; Warren et al., 2020), yet there are a lack of genome-wide association studies linking genetic variants to tau burden and phosphorylation. Furthermore, whole genome sequencing of NHP colonies can identify animals carrying endogenous mutations, which would allow investigators to bypass genetic manipulation for creating models of neurodegeneration and interrogate animals for bona fide pathology. At the Wisconsin National Primate Research Center, we have used this approach in our monkey colonies and identified a family of rhesus macaques carrying a heterozygous *MAPT* R406W mutation. This point mutation, occurring in exon 13, is autosomal dominant in human FTDP-17 and dramatically reduces microtubule binding ability of tau (Dayanandan et al., 1999). Ongoing studies in our lab aim to phenotype these animals using a comprehensive battery of behavioral, imaging, and biochemical measures.

### 8.3 The ethical challenge of modeling tauopathies in NHPs

AD and FTD are tragic diseases that deeply affect patients and their families. The dementia field knows what is needed to advance patient care: early and accurate diagnoses, neuroprotective therapies, and better symptom management, to name a few. Can we afford not to do animal research? As our closest relatives, NHPs can provide answers to help patients. Yet the decision to study these disorders in NHPs must balance the harm vs. benefit to be gained.

A true model of tauopathy may induce behaviors, such as intense anxiety, that require appropriate care to ensure animal health. Therefore, a partnership between researchers and the veterinarian care team is essential. Veterinarians and animal technicians can provide insight into NHP spontaneous activities and managing extreme behaviors with enrichment opportunities and/or medications defined by individual cases. The housing of these animals may require additional steps to ensure a stable environment both within the animal enclosure as well as the animal room or pen; this should be thoroughly discussed with the animal care team. In addition to compatibility between subjects, evaluation of new pairings or social housing must include the emergence or extinction of abnormal behaviors (e.g., stereotypies, self-injury behaviors). Breeding of NHP mutation carriers is envisioned to produce cohorts of affected animals; its success depends on expert pedigree pairing for mating, as well as to protect offspring. *In vitro* fertilization with gametes collected from mutation carriers can facilitate the expansion of a colony but may be limited by veterinarian expertise and institutional commitment to the NHP colony.

NHPs are a limited resource, and access to monkeys is becoming increasingly difficult, which is compounded by the rise in regulatory burden and cost of NHP research. In this context, prioritization of models to be developed and expanded should emphasize their clinical relevance. Tauopathies require time for phenotypic emergence, thus, care must be taken to ensure animal wellbeing and the use of minimally invasive methods of evaluation that will lead to the production of faithful, replicable results. In this context, comprehensive multimodal blind analyses and properly powered studies with aged- and sex-matched control population standards will provide the answers needed to ultimately advance dementia care.

## 9 Conclusion

The study of tauopathies in animals is essential to understand the interaction of multiple biological factors that lead to pathological accumulation of tau, as well as to identify and validate therapeutic targets. NHPs can provide unique insight due to their complex behavior and neurobiology and the similarity of the tau protein across humans and NHPs. Applying rigorous methods that mirror human studies while caring for the animals’ wellbeing will be critical for producing meaningful studies of tauopathies in NHPs.
